# Multi-modal magnetic resonance imaging in a mouse model of concussion

**DOI:** 10.1038/s41597-021-00985-w

**Published:** 2021-08-05

**Authors:** Xuan Vinh To, Fatima A. Nasrallah

**Affiliations:** 1 The Queensland Brain Institute, The University of Queensland, Saint Lucia, Australia; 2 The Centre for Advanced Imaging, The University of Queensland, Saint Lucia, Australia

**Keywords:** Data publication and archiving, Neurodegeneration

## Abstract

This data collection contains Magnetic Resonance Imaging (MRI) data, including structural, diffusion, stimulus-evoked, and resting-state functional MRI and behavioural assessment results, including acute post-impact Loss-of-Righting Reflex time and acute, subacute, and longer-term Neural Severity Score, and Open Field Behaviour obtained from a mouse model of concussion. Four cohorts with 43 3–4 months old male mice in total were used: Sham (n = 14, n = 6 day 2, n = 3 day 7, n = 5 day 14), concussion day 2 (CON 2; n = 9), concussion day 7 (CON 7; n = 10), concussion day 14 (CON 14; n = 10). The data collection contains the aforementioned MRI data in compressed NIFTI format, data sheets on animal’s backgrounds and behavioural outcomes and is made publicly available from a data repository. The available data are intended to facility cross-study comparisons, meta-analysis, and science reproducibility.

## Abstract (summary)

**Table:** 

Measurement(s)	brain measurement
Technology Type(s)	magnetic resonance imaging (MRI)
Sample Characteristic - Organism	Mus musculus

Machine-accessible metadata file describing the reported data: 10.6084/m9.figshare.14813325

## Background & Summary

Concussion or mild traumatic brain injuries (mTBI) account for 70–90% of all Traumatic Brain Injury (TBI)0^1–4^. Existing options for diagnosis of concussion in humans are limited to clinical assessment and exclusion of more severe forms of brain injuries^5^. Most often and by definition, concussions are usually associated with normal findings on conventional routine imaging techniques, which makes objective diagnosis and monitoring using neuroimaging methods difficult. Evidence points to a window of vulnerability after an mTBI before complete recovery where a subsequent impact may cause disproportionate consequences, relative to if the subsequent impact occurs outside of this window^6–8^. Certain sub-populations are at increased risk for repetitive concussion, including sport athletes, military personnel, and domestic violence victims^9^. The inability to accurately and objectively diagnose concussion and define complete recovery post injury, combined with the aforementioned vulnerable sub-populations created the prevalent danger of people being released into risky activities where they are highly likely to receive repetitive concussions and disproportionate cumulative damage. The long-term consequences of concussions—especially repetitive concussion—and the risk of developing dementia, particularly Alzheimer’s disease, has been driving the development of methods that can detect and diagnose changes post-concussion^10,11^. Magnetic Resonance Imaging (MRI) is a non-invasive and non-ionising radiation *in vivo* imaging method with diverse modalities to detect different aspects brain macro- and micro-structure and/or function. MRI can be used for repeated longitudinal imaging measurements, ideal for tracking the temporal trajectory of the recovery process post-concussion.

Another crucial point to note is that concussions are inherently diverse and heterogenous in nature making it difficult to generalise to the wider population. Animal models allow for a more controlled platform where variable factors, such as the severity of the injury and other premorbid effects can be controlled, and the time post-injury can be better accounted for. We employed a model of human concussion by including a rotational force component^12^, similar to the Closed-Head Impact Model of Engineered Rotational Acceleration (CHIMERA), developed by Namjoshi *et al*.^13^.

**Fig. 1:**
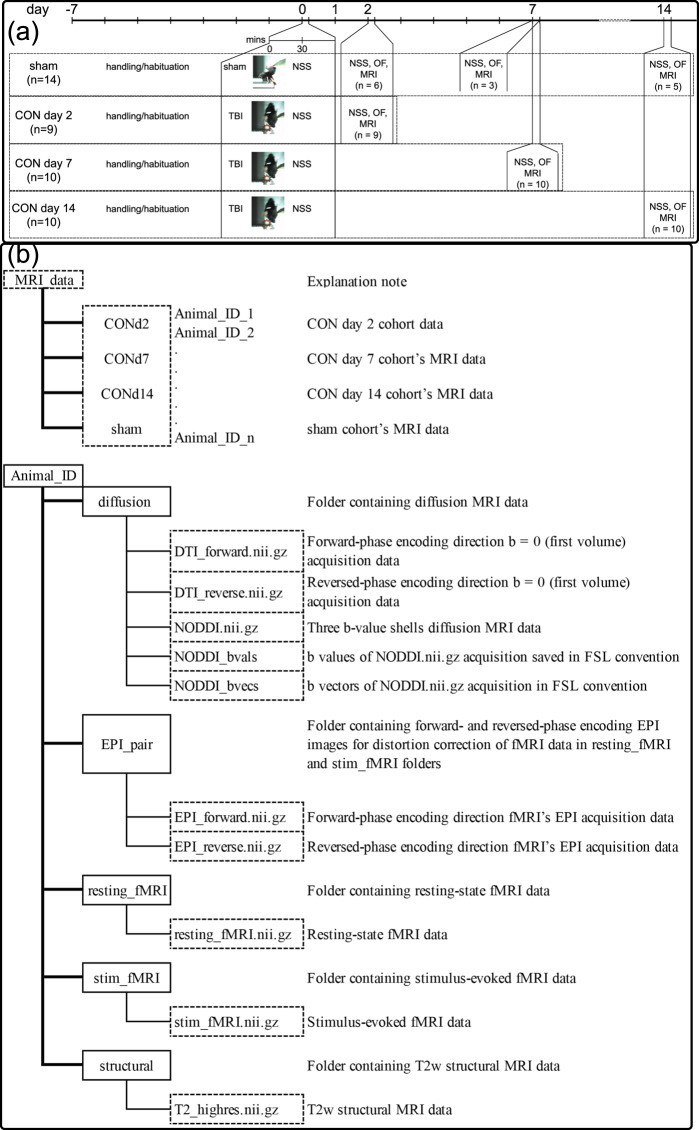
Experimental design and folder and file structure in the collection. (**a**) Summary of the experimental design. CON = Concussed, TBI = Traumatic Brain Injury, NSS = Neural Severity Score, OF = Open Field, MRI = MRI acquisition. (**b**) folder and file structure in the collection.

The data described in this collection was from a cross-sectional behavioural and MRI investigation into the dynamic changes in the concussed animals’ behaviour and brain MRI. The mice were examined at day 2, day 7, and day 14 after a sham or injury procedure for motor-balance symptoms, Open Field behaviour, routine T2-weighted structural imaging, Diffusion Tensor Imaging (DTI), Neurite Orientation Density and Dispersion Imaging (NODDI), stimulus-evoked and resting-state functional Magnetic Resonance Imaging (fMRI). Analysis of behavioural data and MRI data and their relationships in this model have been published elsewhere^14^. This manuscript aims to describe the MRI data generated in more detail and make it available for public access, as well as their acquisition, quality control, and suggested processing/analysis strategy. Overview of the experimental timing can be found in Fig. 1a.

## Methods

The experimental methods described below are expanded versions of descriptions in our related works^14,15^.

### Study design

This manuscript reports the data collection of a cross-sectional study which involved 43 3–4 months old (mice age on procedure date: 13.2 ± 1.4 weeks) male C57BL/6 mice. Mice were randomly assigned into one of the four cohorts: Sham (n = 14, n = 6 day 2, n = 3 day 7, n = 5 day 14), concussion day 2 (CON 2; n = 9), concussion day 7 (CON 7; n = 10), concussion day 14 (CON 14; n = 10). The identity tracking of the mice was done using cage number, permanent marker marks on the animal’s tails, and duplicated identity records on cage cards and lab books. The experimenter was not blinded to the identity of the mice during the whole experiment and MRI analysis procedure.

On day 0, a concussive impact was delivered to the mice in CON groups with our impactor device, which will be described below. The sham animals were put through the exact same procedure without the impact. Immediately after the impact or sham procedure, Loss-of-Righting-Reflex [LRR] time was assessed. Thirty minutes after the recovery of righting reflex, the Neuro Severity Score (NSS) was measured. Depending on the cohorts, at day 2, day 7, or day 14, NSS assessment, Open Field Assessment, and MRI scan were performed on each animal. Obvious brain injuries or structural abnormalities on T2-weighted structural MRI images—including hyper- or hypo-intensity areas and tissue loss—were the exclusion criteria in this study. No animal was excluded from this study. All experiments were approved by the Institutional Animal Ethics Committee at the University of Queensland (Animal Ethics Committee approval number QBI/260/17).

### Impactor device

**Fig. 2:**
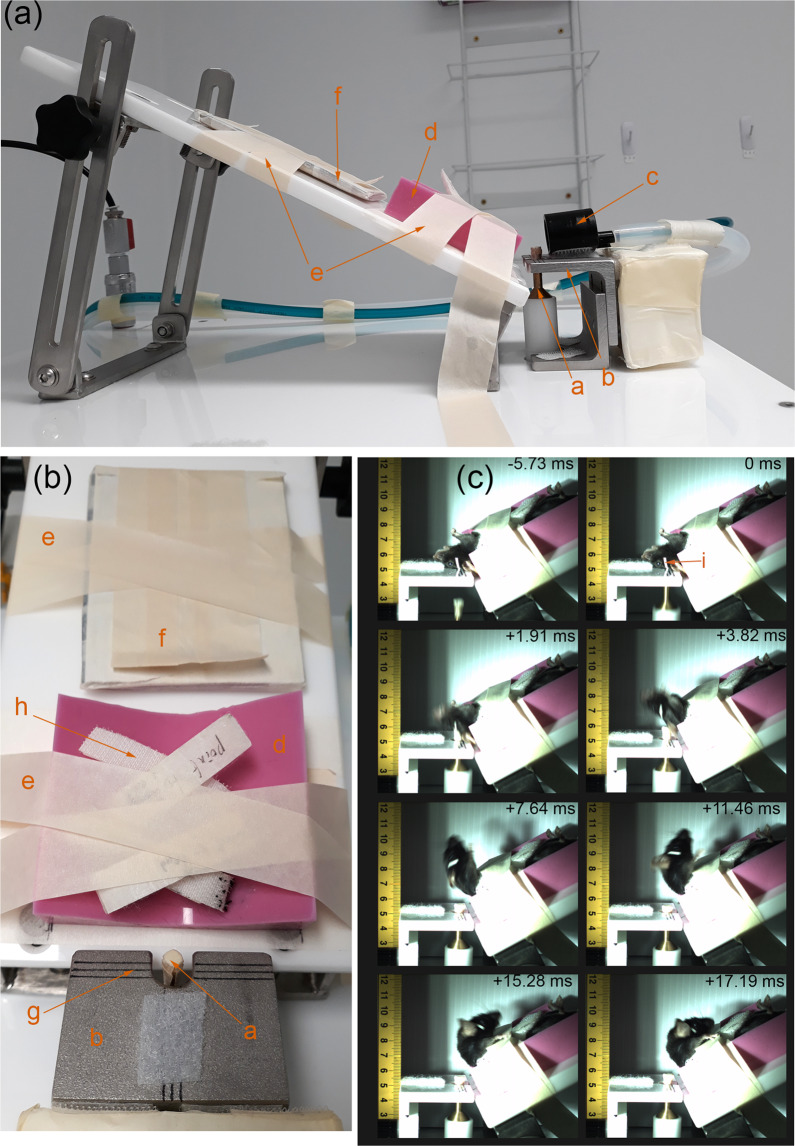
Schematic of the impactor device. (**a**) Side view of the body and head support of the device with the piston at the highest (impact) position, (**b**) Top view the body and head support of the device with the piston at the highest (impact) position and the nose cone (orange c) removed for clarity, (**c**) frame-by-frame breakdown of high-speed camera footage of mouse cadaver’s head impacted at piston velocity = 5.2 m/s from just before the start of the impact and the furthest head position. (Orange letters) a = piston and brass impactor tipped with neoprene rubber, b = head support plate, c = isoflurane anaesthesia nose cone, (**d**) = non-slip silicone body support, (**e**) = masking tape restraints, (**f**) = tail restraint, g = “crosshairs” to “aim” the impactor at head position marker (**i**), h = Velcro body restraint straps, i = 1 mm thick masking tape indicating position to be centred around (**g**) crosshair. Figures and legends previously included in^15^.

Details of the impactor device to produce the injury has been published previously^15^. The impactor was fabricated as a brass piston ending with a 3 mm impactor tip covered with a 2 mm layer of soft neoprene rubber; the total weight of the moving piston was 206.627 g, and the length was 30 cm. The piston is driven by compressed air on one end and vacuum on another. The piston’s motion was stopped by a hard-physical stop, and a solenoid valve was activated to swap the direction of vacuum and compressed air pressure, allowing the piston to quickly retract out of the way before the mouse head drops down. An air pressure regulator and air pressure gauge allowed for reproducible air pressure to be maintained and two laser sensors calculated the terminal piston velocity. The injury device, the important component for positioning the animals’ head and causing the injury, and the captured highspeed camera footage are shown in Fig. 2.

### Impact procedure

The animals were housed in the institutional animal holding facility with a 12-hour light-dark cycle, with *ad libitum* food and water. The animals were conditioned and familiarised to the experimenter for at least seven days prior to the impact/sham procedure by being picked up by the tail and placed on the experimenter’s gloved hands (with the tail secured by the experimenter’s other hand) for two minutes each day. Anaesthesia induction was performed for each animal with 3% isoflurane in 60% Air 40% O_2_ gas mixture at 2 L/min for 2 min in an anaesthesia induction box (sealable box with a gas inlet and another gas outlet to scavenge excess isoflurane). Anaesthetised mice were then secured to the non-slip silicone body support pad (item d on Fig. 2a,b) by four Velcro strips across the mice’s chest and abdomen (item h on Fig. 2b). Anaesthesia during the rest of the positioning procedure was maintained using 2% isoflurane in the same gas mixture and flow rate delivered through a nose cone (item c on Fig. 2a). The non-slip silicone body support pad was positioned on a reclining body support plate. The mice head was supported by a head plate (item b on Fig. 2a,b). The head plate had cut out for a brass piston (item a on Fig. 2a,b) to deliver the impact; a set of lines (item g on Fig. 2b) were used to aim and position the head consistently across different animals. The tail was secured to a stiff panel (item f on Fig. 2a,b). Once the head was correctly positioned, strips of tape (item e on Fig. 2a,b) were used to secure the tail restraint panel and silicone body support to the body support plate.

Once the animal’s body was secured and the head was correctly positioned, the nose cone was removed to discontinue anaesthesia, and the trigger button was immediately pressed to initiate the impact. Piston velocity was calibrated to range from 5.2 to 5.4 m/s. The velocity range corresponded to the “high” impact classification in our earlier publication^15^. Mice in sham group went through the same procedure, just with a mock pressing of the trigger and no impact delivered.

Time under anaesthesia for all animals were 10–12 minutes. This time was kept consistent by the experimenter aligning all experimented animals to the same accuracy standard.

### Behavioural assessment

Details of the Neuro Severity Score (NSS), Open Field Assessment, and MRI experiments, have been described in our earlier publication^14^, reproduced here for clarity and ease for the readers.

#### Loss-of-righting reflex (LRR) assessment

After the impact was delivered or the mock pressing of the trigger, the mice were freed from the tape and Velcro restraints and placed on an electrically warmed surface in the supine position (on their backs) and monitored for signs of recovery. Loss-of-Righting-Reflex (LRR) time was defined as the length of time (in seconds) from the moment of the trigger being pressed (or mock pressed) to the first sign of the animal regaining righting reflex (righting itself to a prone position).

#### Neural Severity Score assessment

Thirty minutes after the recovery of righting reflex, the mice were assessed by a modified Neuro Severity Score (NSS). A second NSS assessment was done on the day of and prior to the MRI scan. The modified NSS assessment was based on Flier *et al*.^16^, where detailed descriptions of the original tasks can be found. These 10 tasks included: successful escape from a 30 cm-diameter walled circle with one opening; natural seeking behaviour in an open area (the same walled circle with the opening closed); lack of limping/dragging walking gait or grabbing weakness (when a pair forceps is place close to one of the mouse’s paws, the mouse will instinctively grab them; lack of such reflex is considered a deficit); presence of a straight walk without an abnormal gait; intact startle response to loud noise (e.g.: experimenter’s hand clap); successful balance for 10 seconds on a 1 cm beam; successful walks across a 30 cm gap on elevated (15 cm above a solid surface) 3, 2, and 1 cm beams (slips and falls off the beams or hesitancy to cross the beam after 2 minutes were considered task failures); and successful balance for 10 seconds on a 0.6 cm round stick. The final task described in Flier *et al*. was the round stick balance task^16^; our preliminary tests showed that some uninjured and unanaesthetised C57Bl/6 mice were sufficiently skilled and motivated walk across the 0.6 mm round stick in a manner similar to other beam walk tasks. Consequently, an additional 0.6 cm round stick walk task was added. The total number of tasks and the highest possible score were raised from 10 to 11. These 11 tasks form the modified NSS assessment.

Failure to perform each of the first five tasks (30 cm circle escape, seeking behaviour, no limping gait or grabbing weakness, straight walk, startle reflex) one point is given. The last six tasks were performed in the sequence described above; a failed task gives one point, and the remaining tasks were skipped and the same number of points as the number of skipped tasks were given.

The animals in this collected had a narrow range of NSS: all animals scored between zero and three NSS. All animals with NSS higher than zero had an NSS of one or two, except one with an NSS of three. All animals with NSS of one failed the 0.6 cm round stick walk task. All animals with NSS of two failed the 0.6 cm round stick balance and walk. The one animal with NSS of three failed the 0.6 round stick balance and walk tasks and the startle reflex task.

#### Open Field assessment

Each animal’s Open field activity was examined on the same day of and prior to the MRI scan. The assessment was performed on an elevated circular platform (77 cm diameter) fenced by 32 cm high transparent boundary. The mice were placed in the centre of the arena and their spontaneous activities were recorded, tracked, standardised, and quantified using an overhead calibrated camera and the Tracker software (Bio-Signal Group, NY, USA) for 10 minutes. The arena was divided into two zones: a central zone consisted of a circular area with the centred around the centre of the area and 48.1 cm diameter and a peripheral zone which consisted of the remaining area of the arena outside the central zone. The thigmotaxis index (TI)—an index for anxiety-like behaviours^17^, was calculated as TI = (T_peripheral_ - T_central_)/(T_peripheral_ + T_central_) where T_peripheral_ and T_central_ represents the time spent in the peripheral and central zone, respectively.

### MRI experiments

MRI acquisitions were performed using a pre-clinical 9.4 T MRI scanner (Bruker Biospin, Germany) equipped with a cryogenically cooled Bruker ^1^H Quadrature transmit/receive MRI CryoProbe™, controlled by a computer running Paravision 6.0.1 software (Bruker Biospin, Germany).

Note on image orientation: while the animals were technically positioned in the “head first, prone” position, the scanning sessions were registered in Paravision 6.0.1 as “head first, supine”. In our experience, this setting makes the orientation displayed on the console intuitive: the animal’s left is on the radiographer’s left and the images are not “upside down”. Acquired MRI data were exported into DICOM format using Paravision 6.0.1 and DICOM images were converted to NIFTI format using MRIcron (i.e., the dcm2nii tool)^18^. The exported NIFTI images, when displayed on FSL (v.6.0.4^19^, https://fsl.fmrib.ox.ac.uk/fsl/fslwiki)’s FSLeyes will have the animal’s left on the viewer’s left, but right on FSLeye’s displayed anatomical labelling. The animal’s anterior-posterior (rostral-caudal) and superior-inferior orientations do not match with FSLeye’s displayed anatomical labelling. This orientation preserves the Z-direction and slice direction in the acquisition and is closest to the raw converted data.

#### Animal handling

*A*naesthesia induction for MRI scan was performed using 3% isoflurane in 60% air, 40% O2 at 1 L/min in an anaesthesia induction box. Once sufficient anaesthesia depth was achieved, the animal was transferred to an MRI-compatible cradle (Bruker Biospin, Germany) and anaesthesia was maintained during the preparation period (30 minutes) using 2–2.5% isoflurane in the same gas mixture and flow rate delivered through a nose cone. Subdermal electrodes were inserted near the 2^nd^ and 4^th^ digits on the left forepaw for mild electrical stimulations, which was delivered by a current source (Isostim A320, World Precision Instrument USA). Respiration rate, respiration pattern, and rectal temperature were monitored by an MRI-compatible physiological monitoring system (Model 1030, SA Instruments Inc, USA). Rectal temperature was maintained at 36.5 ± 0.5 °C via a heated water circulation device (SC100, Thermo Scientific, USA). Ear bars and bite bars were used to fix the animal’s head and reduce head motion

An 18 G needle was used as an initial guide for a polyethylene (PE10, Scientific Commodities Inc., USA) peritoneal catheter to be placed and fixed to the mouse for medetomidine (Domitor, Pfizer, USA) delivery. Upon correct placement of the catheter, the needle guide was withdrawn and fixed in a position outside the scanner bore and the catheter was fixed to the mouse body with surgical tape. Once the animal was placed inside the MRI scanner, a bolus of 0.05 mg/kg medetomidine was given intraperitoneally and a continuous infusion of 0.1 mg/kg/h using a syringe pump was maintained throughout the rest of the scan using a syringe pump. After initiation of medetomidine infusion, Isoflurane concentration was lowered gradually to keep the respiration rate between 100–140 breaths per minute and kept at 0.25% at the lowest throughout the rest of the scan. Each animal was under anaesthesia for 2.25–2.5 hours. Upon the scan completion, 1.25 mg/kg atipemazole (medetomidine reversal) (Antisedan, Pfizer, USA) bolus was given intraperitoneally. Anaesthesia protocol was adapted from Nasrallah *et al*.^20^.

#### Structural MRI scans

Structural imaging data was acquired using a 2D T2-weighted (T2w) Turbo Rapid Acquisition with Refocused Echoes (TurboRARE) sequence with the following parameters: matrix size = 192 × 192, Field of View (FOV) = 19.2 × 19.2 mm, and 52 × 0.3 mm thick slices; giving an output spatial resolution of 0.1 × 0.1 × 0.3 mm, Repetition Time (TR)/Echo Time (TE) = 7200/ 39 ms, averages = 4, RARE factor = 8, bandwidth = 54.3478 kHz, and acquisition time = 5 minutes 27 seconds. The sequence parameter was presented in^14^.

Each animal’s structural MRI scan was converted and saved as compressed NIFTI file named T2_highres.nii.gz inside each animal’s structural folder.

#### Stimulus-evoked and resting-state functional MRI scans

A 2D gradient-echo echo-planar-imaging (GE-EPI) sequence with the following parameters was used for the functional (stimulus-evoked or resting-state) MRI scans: matrix size = 64 × 64, FOV = 19.2 × 19.2 mm, 20 slices of 0.5 mm thickness and 0.1 mm slice gaps; giving an output spatial resolution of 0.3 × 0.3 × 0.6 mm, TR/TE = 1000/14 ms, flip angle = 70°, bandwidth = 200 kHz, fat suppression = ON, FOV saturation (covering the head tissue inferior to the subject’s brain) = ON, and navigator pulses = ON.

Stimulus-evoked fMRI scan was performed, approximately 70 minutes after the initiation of the medetomidine bolus, with the 2D GE-EPI sequence above (400 volumes). Electrical stimulation was delivered based on a block-stimulation design (40 seconds OFF, 20 seconds ON): six OFF-ON blocks, starting at t = 0 second, plus a final 40 seconds OFF block, for a total of 400 seconds (6 minutes 40 seconds). The following parameters were used for the electrical current: 6 Hz pulse frequency, 0.3 ms pulse width, and 0.6 mA current^20^. Stimulus-evoked fMRI data was converted and saved as compressed NIFTI file named stim_fMRI.nii.gz in the stim_fMRI folder inside each animal’s folder.

After the stimulus-evoked fMRI sequence was completed, a 5-minutes break was given prior to the start of the rsfMRI scan. RsfMRI scan was performed with the 2D GE-EPI sequence above (600 volumes) for a total of 600 seconds (10 minutes). rsfMRI data was converted and saved as compressed NIFTI file named resting_fMRI.nii.gz in the resting_fMRI folder inside each animal’s folder. and 600 volumes were acquired

A pair of EPI scans with the same parameters above but with number of averages = 2 and opposite phase-encoding directions (Superior → Inferior then Inferior → Superior) was acquired for distortion correction of the fMRI images. This pair of images was converted and saved as compressed NIFTI files named EPI_forward.nii.gz and EPI_reverse.nii.gz inside each animal’s folder. “Forward” phase means acquisition using the same phase encoding directions as the stimulus-evoked fMRI and rsfMRI. The sequence parameter was presented in^14^.

#### Diffusion MRI scans

Diffusion MRI data were acquired using a Diffusion-Weighted Imaging (DWI) Spin-Echo Echo Planar Imaging (SE-EPI) sequence with the following parameters matrix size = 96 × 96, FOV = 19.2 × 19.2 mm, 32 slices of 0.25 mm thickness and 0.05 mm slice gaps, giving output spatial resolution of 0.2 × 0.2 × 0.3 mm, TR/TE = 4500/25 ms, averages = 4, 3 b-value shells with b = 600, 1500, and 2000 s/mm^2^, diffusion gradient duration = 4.5 ms, diffusion gradient separation = 10.5 ms, 33 diffusion weighted directions for each shell, and 2 b = 0 images. Diffusion MRI sequence acquisition time was 15 minutes and 9 seconds. The multi-shell diffusion data converted and saved as compressed NIFTI file named NODDI.nii.gz inside each animal’s diffusion folder. b values and b vectors information were saved in NODDI_bvals and NODDI_bvecs text files, respectively, (in the FSL convention) in each animal’s diffusion folder.

A pair of reference b = 0 SE-EPI scans were acquired with opposite phase-encoding directions for EPI distortion correction. Acquisition time for each of these reference SE-EPI scans was 32 seconds. This pair of images was converted and saved as compressed NIFTI files named DTI_forward.nii.gz and DTI_reverse.nii.gz inside each animal’s folder. “Forward” phase means acquisition using the same phase encoding directions as the multi-shell diffusion data. Note: the DTI_forward.nii.gz and DTI_reverse.nii.gz images each contain one b = 0 volume and one diffusion-weighted volume; for the purpose of distortion correction, only the b = 0 volume (the first volume in each file) is needed.

The sequence parameter was presented in^14^.

## Data Records

The data has been deposited in a publicly available data repository with the following Digital Object Identifier (DOI): 10.14264/aae348e^21^.

The records of each animals’ Animal ID, LRR time, NSS 30-minutes post-impact and on scanning day are kept in the Animal_records_behavioural-data.xlsx Excel file. This Excel file is divided into four individual sheets, one for each cohort. Each cohort’s sheet is saved as a comma-separated-value file: Animal_records_behavioural-data_ < cohort name > .csv.

Folders structure for the MRI_data folder and the folders and files structure for each animal’s MRI data are described in Fig. 1b and the MRI_data_folder_structure.pdf file.

## Technical Validation

Reproducibility of behavioural MRI data and fitting for this data collection was performed in general by randomly dividing the sham cohort into two pseudo-groups and two sample t-tests were performed on the MRI metrics comparing the pseudo-groups. The specific divisions of the sham cohort’s animals into the two pseudo-groups for the technical validation results of MRI data is shown in Supplementary File 1 and in the sham_reproducibility_group.xlsx Excel sheet and the sham_reproducibility_group.csv comma-separated-value file.

### Behavioural assessments

Two sample unpaired t tests comparing LRR time, NSS at 30 minutes after sham procedure, NSS on scan day, and TI on scan day of the two sham pseudo-groups were conducted. No measures showed significant different between the two sham pseudo-groups. LRR time: p value = 0.506, 95% Confidence interval of difference = −15.5–8.1 seconds. NSS at 30 minutes: p value = 0.218, 95% Confidence interval of difference = −1.1–0.3. NSS on scan day: p value = 0.27, 95% Confidence interval of difference = −0.8–0.2. TI on scan day: p value = 0.54, 95% Confidence interval of difference = −0.225–0.126.

### Structural MRI data

**Fig. 3:**
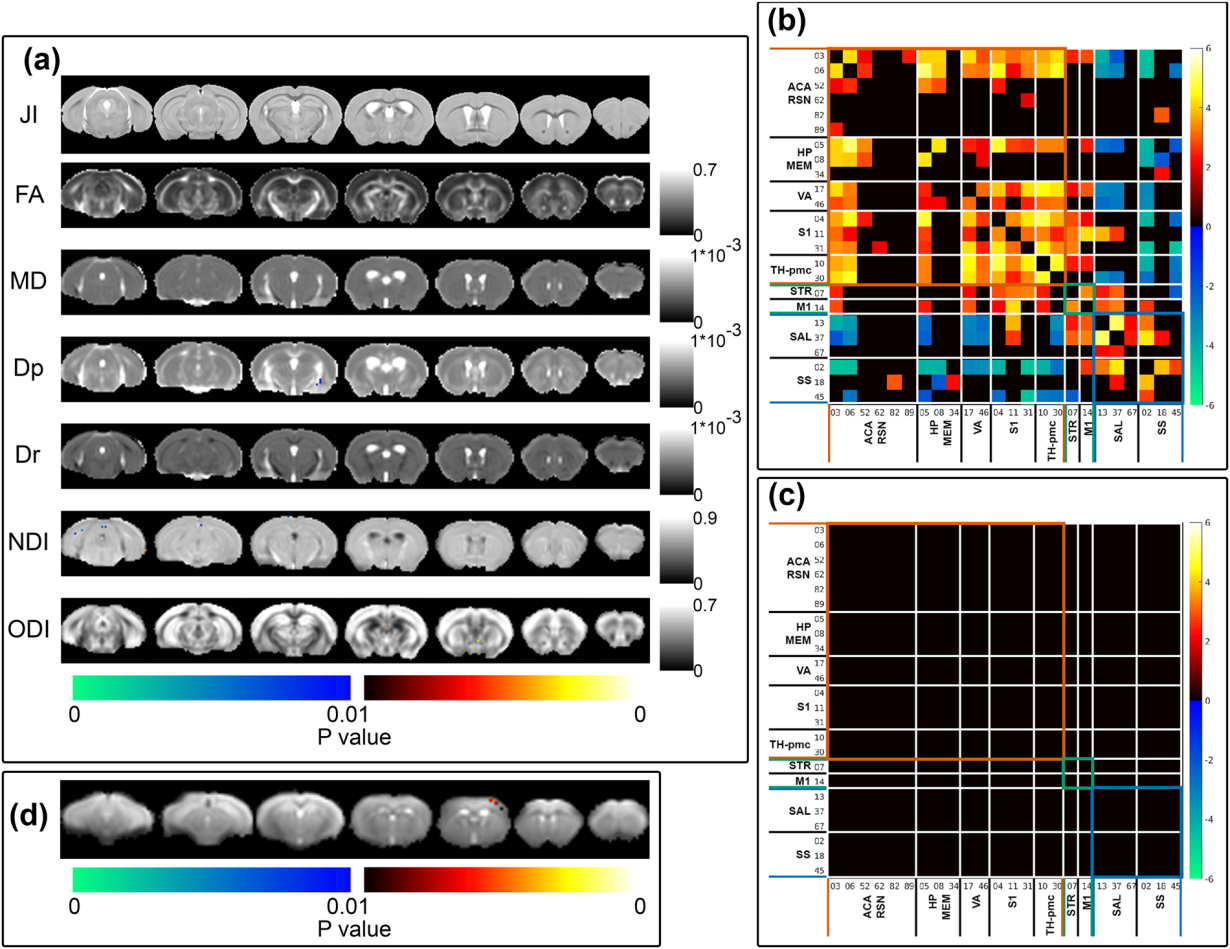
Randomise reproducibility test of MRI data. (**a**) Voxel-by-voxel statistical analysis results of Tensor-based morphometry (JI = Jacobian Index) Diffusion Tensor Imaging (FA = Fractional Anisotropy, MD = Mean Diffusivity, Dp = Parallel Diffusivity, Dr = Radial Diffusivity) and Neurite Orientation Dispersion and Density Imaging metrics (NDI = Neurite Density Index, ODI = Orientation Dispersion Index). Statistical map thresholded at p value < 0.01 (one-tailed), unpaired two sample t-test, implemented as permutation tested for the General Linear Model, corrected for multiple comparisons with mass-based FSL’s Threshold-free Cluster enhancement (TFCE). Statistical maps were overlaid on the averaged and registered structural images, DTI and NODDI metrics maps corresponding to the statistical maps (JI, DTI and NODDI results). Corresponding grey scale map for each averaged DTI and NODDI metrics maps were provided; units for Dp, Dr, and MD were in mm^2^/s. (**b**) One sample t-test result of component-component functional network connectivity across all sham animals (n = 14) and (**c**) Two sample t-test result of component-component functional network connectivity differences between sham cohort’s pseudo-groups. Colour map scaled by z test statistics; non-black cells were defined as component-component functional connectivity deemed statistically significant in permutation-based inference of the General Linear model, corrected for multiple comparison with False Discovery Rate, and thresholded at q value < 0.05 (two-tailed). (**d**) two-sample t-test results comparing sham cohort’s two pseudo-groups stimulus-evoked activation independent component spatial map, implemented as permutation tested for the General Linear Model, corrected for multiple comparisons with mass-based FSL’s Threshold-free Cluster enhancement (TFCE), and thresholded at p value < 0.01 (one-tailed). Figure legends previously included in^14^.

Structural MRI data’s intensity inhomogeneity correction (through FSL’s FAST), intensity-based masking, iterative image registration-template construction using FSL’s FLIRT and FNIRT, and calculation of affine-transformation-included Jacobian Index (JI) were performed as described under the Usage Note, Structural MRI data. Two sample t-test comparing sham cohort’s pseudo-groups’ JI was performed using permutation inference for the General Linear model^22^ as implemented in FSL’s randomise^23^; the number of permutation was set to 10000. Statistical map was corrected for multiple comparisons with mass-based FSL’s Threshold-free Cluster enhancement (TFCE)^24^ and thresholded at p-value < 0.01 (one-tailed). Structural MRI and Tensor-based morphometry reproducibility and reliability test result is shown in Fig. 3a, JI. No systemic difference in JI between the two pseudo-groups were detected.

### Diffusion MRI data

Diffusion MRI data’s EPI distortion correction, DTI and NODDI fitting, and image registration were performed as described under the Usage Note, Diffusion MRI data. Two sample t-tests comparing the pseudo-groups’ DTI and NODDI metrics were performed using permutation inference for the General Linear model^22^ as implemented in FSL’s randomise^23^; the number of permutation was set to 10000. Statistical maps were corrected for multiple comparisons using mass-based FSL’s Threshold-free Cluster enhancement (TFCE)^24^ and thresholded at p-value < 0.01 (one-tailed). Diffusion MRI and DTI/NODDI fitting reproducibility and reliability test results are shown in Fig. 3a (FA, MD, Dp, Dr, NDI, ODI). No systemic patterns of differences in DTI and NODDI metric maps between the two sham pseudo-groups were detected.

### Stimulus-evoked functional MRI data

Stimulus-evoked fMRI data’s slice-timing correction, de-spiking, motion correction, distortion correction, band-pass filtration (at 0.01–0.3 Hz), and iterative image registration/template construction were performed as described under the Usage Note, Functional MRI data. Artefact removal from pre-processed and registered stimulus-evoked fMRI data were performed with Group Information Guided Independent Component Analysis (GIG-ICA)^25^, as implemented in the Group ICA of fMRI Toolbox (GIFT)^26^. The number of independent component was set to 50. Artefactual components were classified based on criteria similar to prior publications^27,28^: (1) component spatial map having a large overlap with white matter (WM) tracts and/or the ventricles (2) spatial maps with ring-like or crescent shape patterns around the brain-air boundary or regions susceptible to EPI distortion, (3) frequency power spectrum showing pan-frequency distribution, (4) component spatial maps with alternating positive and negative correlating bands.

Artefact-cleaned stimulus-evoked fMRI data was then decomposed into independent components using Infomax^29,30^ temporal-concatenated spatial group ICA. The number of components was set to 18 and an independent component whose group-level spatial maps contained the primary somatosensory cortex, which was shown to be activated in the mouse brain in stimulus-evoked fMRI^20^, and component time course similar to the stimulation paradigm delivered was chosen for further analysis using the pseudo-group design. Two-sample t-test comparing the two pseudo-groups’ individual-level spatial maps of the primary somatosensory cortex independent component was performed using permutation inference for the General Linear model^22^ as implemented in FSL’s randomise^23^; the number of permutation was set to 10000. Statistical maps were corrected for multiple comparisons using mass-based FSL’s Threshold-free Cluster enhancement (TFCE)^24^ and thresholded at p-value < 0.01 (one-tailed). The result is shown in Fig. 3d.

### Resting-state functional MRI data

RsfMRI data’s slice-timing correction, de-spiking, motion correction, distortion correction, band-pass filtration (at 0.01–0.3 Hz), and iterative image registration/template construction were performed as described under the Usage Note, Functional MRI data. Artefact removal was performed with Group Information Guided Independent Component Analysis (GIG-ICA)^25^, as implemented in the Group ICA of fMRI Toolbox (GIFT)^26^, decomposing the data to 50 Independent Components. Artefactual components were classified based on criteria similar to that of stimulus-evoked fMRI described above.

Artefact-cleaned rsfMRI data were decomposed into resting-state functional independent components using combined Independent Vector Analysis (IVA-GL): combining IVA with multivariate Gaussian source component vectors (IVA-G)^31^ and IVA with Laplace source component vectors (IVA-L)0^32^. Group-averaged Independent Components’ (ICs) spatial maps were generated, scaled, and converted to Z-scores, then thresholded at Z > 1. The ICs were classified into functionally-relevant components based on the following criteria^27,28^: (1) component spatial maps occupying spatially and functionally feasible grey matter, (2) frequency power spectrum being dominated by power in the 0.01–0.1 Hz range more than in the 0.1–0.3 Hz range, (3) component spatial maps have little or no overlap with white matter (WM) tracts and ventricle areas, (4) component spatial maps with bilateral or midline-only patterns, (5) if an IC’s spatial map has a unilateral pattern; it was only included if another IC had a contralateral pattern spatial map, (6) ICs were excluded if their spatial maps had a thickness in the rostral-caudal direction equivalent to one EPI slice in the acquisition resolution (these were likely to be single-slice artefacts). Twenty-four ICs passed the criteria and their spatial maps, time courses, and frequency power spectra are shown in Supplementary File 2. The “functionally relevant” components were consistent with prior publications^27,28^.

One sample t-test across all sham animals and two sample t-test comparing two sham pseudo-groups’ component-component functional network connectivity were performed using permutation inference for the General Linear model^22^ as implemented in FSL’s randomise^23^; the number of permutation was set to 10000. Multiple comparison corrections on the results were performed using False Discovery Rate at q value < 0.05. One sample t test result is shown in Fig. 3b, demonstrating significant long-range functional connectivity and the connectivity architecture can be classified into large networks and supra-networks with distinct characteristics^14^. Two sample t-test of the pseudo-groups component-component functional connectivity (Fig. 3d) shows no difference between the pseudo-groups.

## Usage Notes

Detailed data processing and analysis procedures, and the results for the structural, diffusion, and stimulus-evoked and resting-state functional MRI data were described in our previous publication^14^. In this section, we describe some of specific details and issues and our experience handling the data. Some, though not all of the usage notes described below, are expanded versions of processing methods used in our related work^14^. In general, rodent MRI data were given a header file with voxel size 20 times larger than the original size as adaptation to image processing tools originally developed for handing human brain-sized images^33^. More detailed information on the usage of the image processing tools can be found in their respective documentations.

### Structural MRI data

Voxel-wise morphometry analysis on mice brain without obvious structural deficits and injuries (such as tissue loss, hyper- or hypo-intensity areas) can be performed without much complication using non-linear image registration-based Tensor-based morphometry^14,34^. Structural signal inhomogeneity correction can be performed on T_2_w structural data using FSL’s FAST tool (https://fsl.fmrib.ox.ac.uk/fsl/fslwiki)^19^ or N4ITK bias field correction^35^ as implemented in Advanced Normalisation Tool (ANTs v.2.3.4)0^36^. We had good image registration results without skull-stripping so this step was skipped in favour of a simple masking step by zeroing all voxels in the image below the 22^nd^ percentile in our previous publication^14^. Nevertheless, skull-stripping for rodents can be performed using 3D Pulse-Couple Neural Networks (3D PCNN)^37^.

We had achieved good registration results using an iterative image-registration and study-specific template construction described previously^14^. Briefly, the structural image was intensity normalised to have the same global averaged intensity across all subjects then registered rigidly using FSL’s FLIRT^19^ to a resampled isotropic 0.1 mm resolution Australian Mouse Brain Mapping Consortium (AMBMC, www.imaging.org.au/AMBMC) MRI template. The registered images from all animals were combined and averaged to create a study-specific structural template. This study-specific template was used as the reference template for another iteration image registration and template creation. This iterative process was repeated for three linear registration iterations and two non-linear registration, using FSL’s FNIRT tool^19^. flirt and fnirt commands’ parameters were as specified in ---flirt and fnirt commands ---in Box 1.

Affine-transformation-included Jacobian determinant (T2highres_ < template > _jac_withaff) can be calculated from the non-linear registration warping field with the command under ---fnirtfileutils commands--- in Box 1.

Alternatively, the iterative registration and study-specific template generation has been automated in ANTS’s^38^ antsMultivarateTemplateConstruction2.sh. The settings for the script found to be suitable for this study’s data can be found under ---ANTS build template--- in Box 1:

Box 1 Structural MRI processing commands. ---flirt and fnirt commands---

flirt -in T2_highres.nii.gz -ref <template> -out T2highres_<template>_FLIRT -omat T2highres_<template>_FLIRT.mat -searchrx −45 45 -searchry −45 45 -searchrz −45 45 -dof 12 -interp spline -cost corratio -searchcost corratio

fnirt --ref=<template> --in = T2_highres.nii.gz --refmask=<template>_mask --inmask=T2_highres_mask.nii.gz --aff=T2_highres_template_FLIRT.mat --iout=T2highres_<template>_FNIRT --cout= T2highres_<template>_FNIRT_warp --warpres=4,4,6 --splineorder=3 --intmod=none --biasres=18,18,18 --intorder=5 --subsamp=4,4,4,4,4,2,2,2,2,1 --miter=6, 6, 6, 6, 6, 6, 6, 6, 6, 6 --reffwhm=0, 0, 0, 0, 0, 0, 0, 0, 0, 0 --infwhm=0, 0, 0, 0, 0, 0, 0, 0, 0, 0 --lambda=960, 640, 480, 320, 240, 240, 160, 120, 80, 60 --estint=0, 0, 0, 0, 0, 0, 0, 0, 0, 0 --applyrefmask=1, 1, 1, 1, 1, 1, 1, 1, 1, 1 --applyinmask=1, 1, 1, 1, 1, 1, 1, 1, 1, 1 --verbose --ssqlambda=1

# <template> in here refers to either the AMBMC mouse brain template or the different iterations of the study specific template.

---fnirtfileutils command---

fnirtfileutils --in = T2highres_<template>_FNIRT_warp --ref = T2highres_<template>_FNIRT_warp --withaff --jac=T2highres_<template>_jac_withaff

# T2highres_<template>_jac_withaff is the Affine-transformation-included Jacobian determinant.

---ANTs build template---

antsMultivariateTemplateConstruction2.sh -d 3 -b 1 -c 2 -g 0.2 -i 10 -j 16 -k 1 -q 320 × 160 × 160 × 80 × 40 × 20 × 20 × 20 × 20 × 20 -f 4 × 2 × 2 × 1 × 1 × 1 × 1 × 1 × 1 × 1 -s 2 × 2 × 1 × 0 × 0 × 0 × 0 × 0 × 0 × 0 -n 1 -o <study_template> -m MI input_images.txt

# <study_template> is the name of the output study-specific template.

### Functional MRI data

We performed slice-timing correction using FSL’s slicetimer tool and despiking of data using AFNI’s 3dDespike (https://afni.nimh.nih.gov/) tool^14^. The usage of these tools did not require special fine-tuning and more information on their usage can be found on the respective tools’ documentation.

For EPI distortion correction using a pair of opposite phase-encoding directions scan, the forward-phase and reverse-phase images should be acquired consecutively, minimising potential frequency and spatial drifts. The functional MRI data should be motion-corrected with the forward-phase EPI image as the target; this is so that the unwarping field calculated can be directly applied to the motion-corrected functional MRI data. FSL’s MCFLIRT was used for this purpose^14^ and the parameters can be under ---mcflirt command--- in Box 2:

Distortion correction of EPI data can be performed for this collection’s functional MRI data using opposite phase-encoding directions EPI data and FSL’s TOPUP^39,40^. The parameters of the TOPUP command for functional MRI distortion correction found to be suitable for this study’s data is given under ---topup command--- in Box 2.

Functional MRI images can be undistorted using the using applytopup command under ---applytopup command--- in Box 2

Distortion corrected EPI images’ intensity inhomogeneity can be corrected using N4ITK bias field correction^35^ as implemented in Advanced Normalisation Tool (ANTs v.2.3.4)^36^, using the command under ---N4BiasFieldCorrection command--- in Box 2.

Image registration for fMRI data can be performed using an iterative direct EPI-to-EPI non-linear registration/template generation process described in our earlier publication^14^, reproduced here. All subject’s distortion corrected EPI images were affinely registered to one subject’s EPI image chosen as reference using FSL’s FLIRT^19^; the registered EPI images were combined and averaged to create the first iteration of an EPI template. This process was then repeated once or several more times with linear registration or with non-linear registration using FSL’s FNIRT tool instead^19^, and subsequent iterations of EPI template were created. For the purpose of group analysis in functional MRI studies, traditional step-wise registration from EPI image to same-subject structural image, followed by registration of structural image to common space structural template can be substituted by direct EPI-to-EPI non-linear registration^41^. Specifications for the FLIRT and FNIRT command found to be suitable for this study’s data is given under ---flirt and fnirt commands--- in Box 3.

Further analysis of stimulus-evoked and resting-state fMRI can be performed in a number of ways, for example, using General Linear Model as implemented in FSL’s FEAT^42,43^, or more data-driven approaches such as Group Independent Component Analysis as implemented in FSL’s MELODIC^44,45^ or the the Group ICA of fMRI Toolbox (GIFT)^26^, or Independent Vector Analysis^31,32^ also implemented in GIFT.

Box 2 Motion and distortion correction commands for functional MRI images. ---mcflirt command---

mcflirt -in fMRI -o mc_fMRI -cost mutualinfo -dof 6 -reffile EPI_forward.nii.gz -stages 4 -verbose -sinc_final

# fMRI refers to the slice-timing corrected and despiked data (as described above). mc_fMRI refers to the motion-corrected data. -reffile field specified the reference image that the registration process of mcflirt will aim to align all volumes of the image specified with -in (fMRI) with. In this case, all functional data that will rely on the forward and reverse acquisition pair for distortion correction should be aligned through motion correction to the forward-phase acquisition image, which is EPI_forward.nii.gz for functional data.

---topup command---

fslmerge -t EPI_fr EPI_forward.nii.gz EPI_reverse.nii.gz

topup --imain = EPI_fr --datain = TOPUP_datain_fMRI --out = cEPI_FR --iout = cEPI_FR_example --warpres = 6 --subsamp = 4,2,1 --fwhm = 0,0,12 --miter = 200,200,200 --lambda = 1e-08,1e-08,1e-08 --ssqlambda = 1 --estmov = 0 --minmet = 0,0,0 --scale = 1 --splineorder = 3 --regrid = 0 --verbose

# TOPUP_datain_fMRI refers to a text file that contains information on how the volumes in --imain was acquired. For this data set, this file contains:

0 -1 0 0.00032

0 1 0 0.00032

# The −1 and 1 in the second column described the phase-encoding directions in the -y and + y directions, respectively. The numbers in the fourth column are the total readout time.

# cEPI_FR is the output unwarping field

---applytopup command---

applytopup --imain = mc_fMRI --topup = cEPI_FR --datain TOPUP_datain_fMRI --inindex = 1 --out = cmc_fMRI --method = jac

# mc_fMRI refers to the motion corrected fMRI data (the output of the mcflirt command above), and cmc_fMRI is the distortion corrected and motion corrected fMRI data.

---N4BiasFieldCorrection command---

N4BiasFieldCorrection -d 3 -i cEPI_FR_example.nii.gz -s 1 -c [400x400x400x400,0] -t [0.15,0.01,256] -v 1 -o [N4cEPI_FR_example.nii.gz,N4cEPI_FR_example_bias.nii.gz]

# N4cEPI_FR_example refers to the bias field and distortion corrected EPI image and N4cEPI_FR_example_bias here refers to the corresponding bias field.

Box 3 Image registration for functional MRI images. ---flirt and fnirt commands---

flirt -in N4cEPI_FR_example.nii.gz –ref <EPI_template> -out N4cEPI_<template>_FLIRT6dof -omat N4cEPI_<template>_FLIRT6dof.mat -dof 6 -searchrx −20 20 -searchry −20 20 -searchrz −20 20 -searchcost mutualinfo -cost mutualinfo

flirt -in N4cEPI_FR_example.nii.gz –ref <EPI_template> -out N4cEPI_<template>_FLIRT -init N4cEPI_<template>_FLIRT6dof.mat -omat N4cEPI_<template>_FLIRT.mat -dof 12 -searchrx −20 20 -searchry −20 20 -searchrz −20 20 -searchcost mutualinfo -cost mutualinfo

fnirt --ref = <EPI_template> --in = N4cEPI_FR_example.nii.gz --aff = N4cEPI_<template>_FLIRT.mat --iout = N4cEPI_<template>_FNIRT --cout = N4cEPI_<template>FNIRT_warp --warpres = 6,6,6 --splineorder = 3 --intmod = global_non_linear --biasres = 40,40,40 --intorder = 5 --subsamp = 4,4,4,4,2,2,2,2,2,1,1,1 --miter = 10,10,10,10,10,10,10,10,10,10,10,10 --reffwhm = 0,0,0,0,0,0,0,0,0,0,0,0 --infwhm = 0,0,0,0,0,0,0,0,0,0,0,0 --lambda = 15000,7500,3750,1875,1875,1250,840,560,375,560,450,375 --estint = 1,1,1,1,1,1,1,1,1,1,1,1 --applyrefmask = 0,0,0,0,0,0,0,0,0,0,0,0 --applyinmask = 0,0,0,0,0,0,0,0,0,0,0,0 --verbose --ssqlambda = 1

# N4cEPI_<template> FNIRT_warp refers to the warping field that can be used to warp the motion corrected and distortion corrected functional MRI images to a common space.

### Diffusion MRI data

Diffusion MRI data of this collection can be corrected for eddy currents and movements using FSL’s eddy_correct tool with no change in parameters; refer to the relevant documentation on eddy_correct usage.

Eddy current and motion corrected diffusion MRI data can be can then distortion corrected using similar procedures as that of the functional MRI data. The specific command for diffusion EPI data is given under ---topup command--- in Box 4.

Note: FSL’s v.5.0.10 and later eddy tool^46,47^ that combine eddy current correction, motion correction, and distortion correction requires a set of diffusion directions that span the entire sphere and not just a half-sphere. The data acquired and presented in this collection had only a half-sphere set of diffusion directions and thus the eddy tool will not be suitable.

Distortion corrected diffusion MRI data can be analysed using Diffusion Tensor Imaging implemented in FSL’s DITFIT, Diffusion Kurtosis Imaging (DKI) implemented in DKI MATLAB toolbox (https://cai2r.net/resources/software/diffusion-kurtosis-imaging-matlab-toolbox)^48^, or the Neurite Orientation Dispersion and Density Imaging NODDI MATLAB Toolbox (https://www.nitrc.org/projects/noddi_toolbox)^49,50^. For this data collection’s NODDI fitting and analysis presented previously^14^, intra-neurite diffusion was modelled as diffusion in cylinders with zero radius and there was no diffusional exchange within the cylinders with the a homogeneous background “cells” compartment; the neurite “cylinders’” orientation was modelled using Watson’s distribution, and the NODDI algorithm used Szafer, *et al*.’s tortuosity model for randomly packed cylinders^51^. Fixed intrinsic diffusivity were set to 2 × 10^−9^ m^2^/s and fixed isotropic diffusivity were set to 4 × 10^−9^ m^2^/s.

Distortion corrected b = 0 image can be corrected for intensity inhomogeneity using N4ITK bias field correction^35^ as implemented in Advanced Normalisation Tool (ANTs v.2.3.4)^36^, using the command under ---N4BiasFieldCorrection command--- in Box 4.

The individual N4cDTI_b0.nii.gz images can be registered through a similar direct EPI-to-EPI iterative image registration and study-specific template creation procedure as described above for functional MRI data. The resulting warping fields can then be used to warp the diffusion metric maps to a common space. We found the following specification works well for ANTS’s^38^ antsMultivarateTemplateConstruction2.sh for this study’s diffusion MRI data as under ---ANTS build template--- in Box 4.

Box 4 Diffusion MRI processing commands. ---topup command---

fslmerge -t DTI_b0_FR DTI_b0_forward DTI_b0_reverse

topup --imain = DTI_b0_FR --datain = TOPUP_datain_EPI --out = cDTI_b0 --iout = cDTI_b0_example --warpres = 8 --subsamp = 4,2,1 --fwhm = 0,0,4 --miter = 50,50,50 --lambda = 1e-8,1e-9,1e-10 --ssqlambda = 1 --estmov = 0 --minmet = 0,0,0 --scale = 1 --splineorder = 3 --regrid = 0 --verbose

---N4BiasFieldCorrection command---

N4BiasFieldCorrection -d 3 -i cDTI_b0_example.nii.gz -s 1 -x cDTI_mask.nii.gz -c [80 × 80 × 80 × 80,0.0001] -t [0.15,0.01,256] -v 1 -o [N4cDTI_b0.nii.gz,N4cDTI_b0_bias.nii.gz]

# cDTI_mask was a mask generated by zeroing all voxels below the 15th intensity percentile of the S0 image (generated by FSL’s DTIFIT).

---ANTS build template---

antsMultivariateTemplateConstruction2.sh -d 3 -b 1 -c 2 -g 0.2 -i 6 -j 12 -k 1 -q 1600 × 800 × 400 × 200 × 100 -f 4 × 2 × 2 × 1 × 1 -s 2 × 2 × 1 × 0 × 0 -n 1 –o <study_template> −m MI -v 8 input_images.txt

# <study_template> refers to the name of the output study specific template.

## Supplementary information

Supplementary File 1

Supplementary File 2.

## Data Availability

Details of software packages and specific versions used were indicated in the Usage Note section. Suggestion of their usage and specification for each command was also incorporated in the Usage Note.
