# Supplementary material for: Multi-modal magnetic resonance imaging in a mouse model of concussion
Source: Sci Data. 2021 Aug 5;8:207. doi: 10.1038/s41597-021-00985-w (PMC8342546; doi:10.1038/s41597-021-00985-w)
Supplement: Supplementary file 1 [file 41597_2021_985_MOESM1_ESM.pdf]

Supplementary File 1: sham reproducibility test pseudo-groups details

Animal ID	Pseudo-group 1	Pseudo-group 2
C1-1s_shamd2	1	0
C3-4s_shamd2	0	1
C4-3s_shamd2	0	1
C4-4s_shamd2	1	0
C5-2s_shamd2	0	1
C9-2s_shamd7	0	1
C9-4s_shamd2	1	0
C10-2s_shamd7	0	1
C11-1s_shamd7	1	0
C12-1s_shamd14	1	0
C12-5s_shamd14	1	0
C13-10s_shamd14	0	1
C14-12s_shamd14	1	0
C14-14s_shamd14	0	1

"1" denote the animal belonging to the group and "0" denote its absence. The number array will also serve as FSL's fsl_glm design matrix.
