# Supplementary material for: Multi-modal magnetic resonance imaging in a mouse model of concussion
Source: Sci Data. 2021 Aug 5;8:207. doi: 10.1038/s41597-021-00985-w (PMC8342546; doi:10.1038/s41597-021-00985-w)

Supplementary File 2: "Signal" resting-state Group Independent Vector Analysis components. Figure previously included in.[1]

1. To, X. V. & Nasrallah, F. A. A roadmap of brain recovery in a mouse model of concussion: insights from neuroimaging. *Acta Neuropathol. Commun.* **9**, 2 (2021).

Anterior Cingulate Area + Prelimbic area

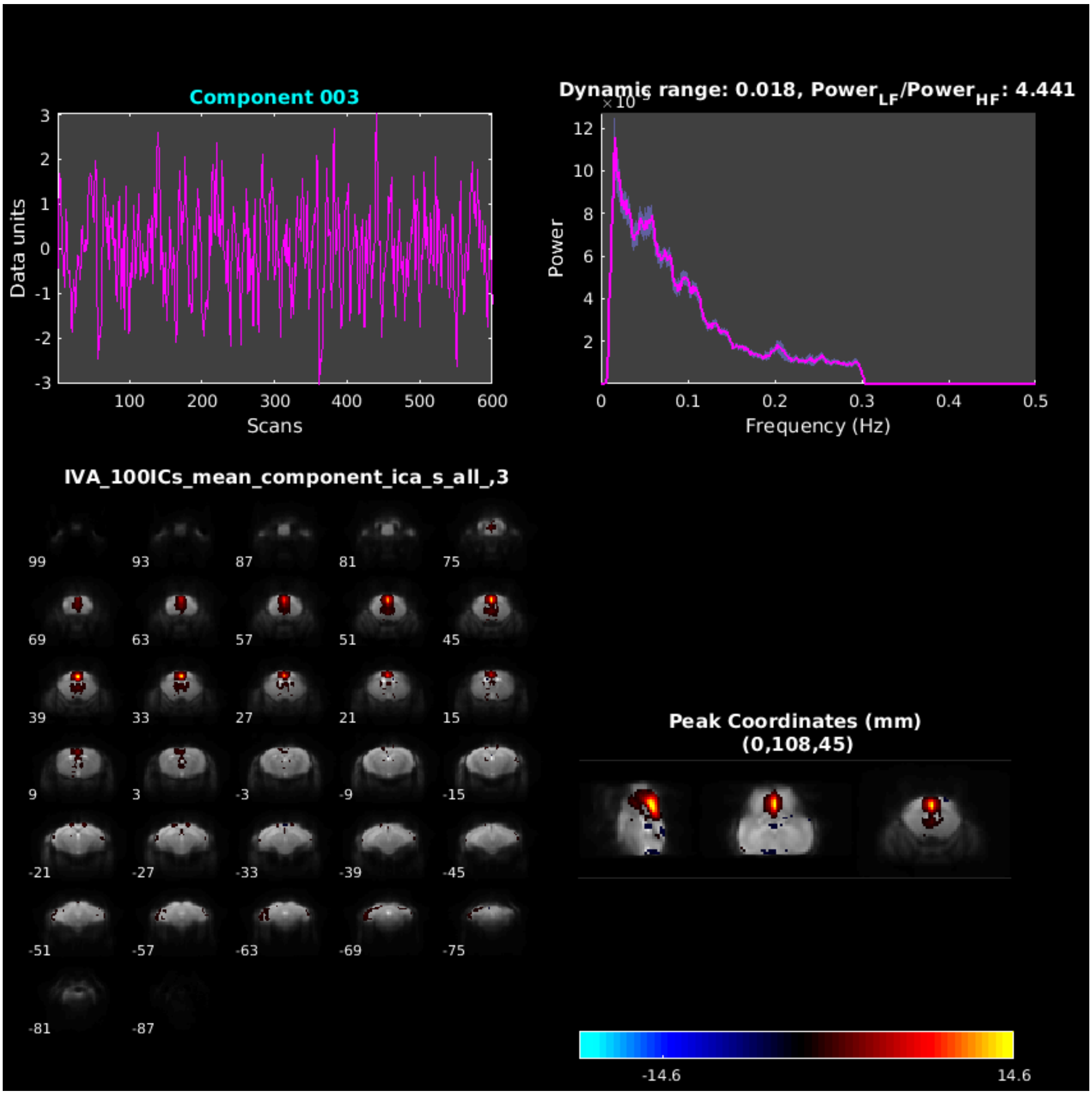

Retrosplenial area + Anterior Cingulate Area

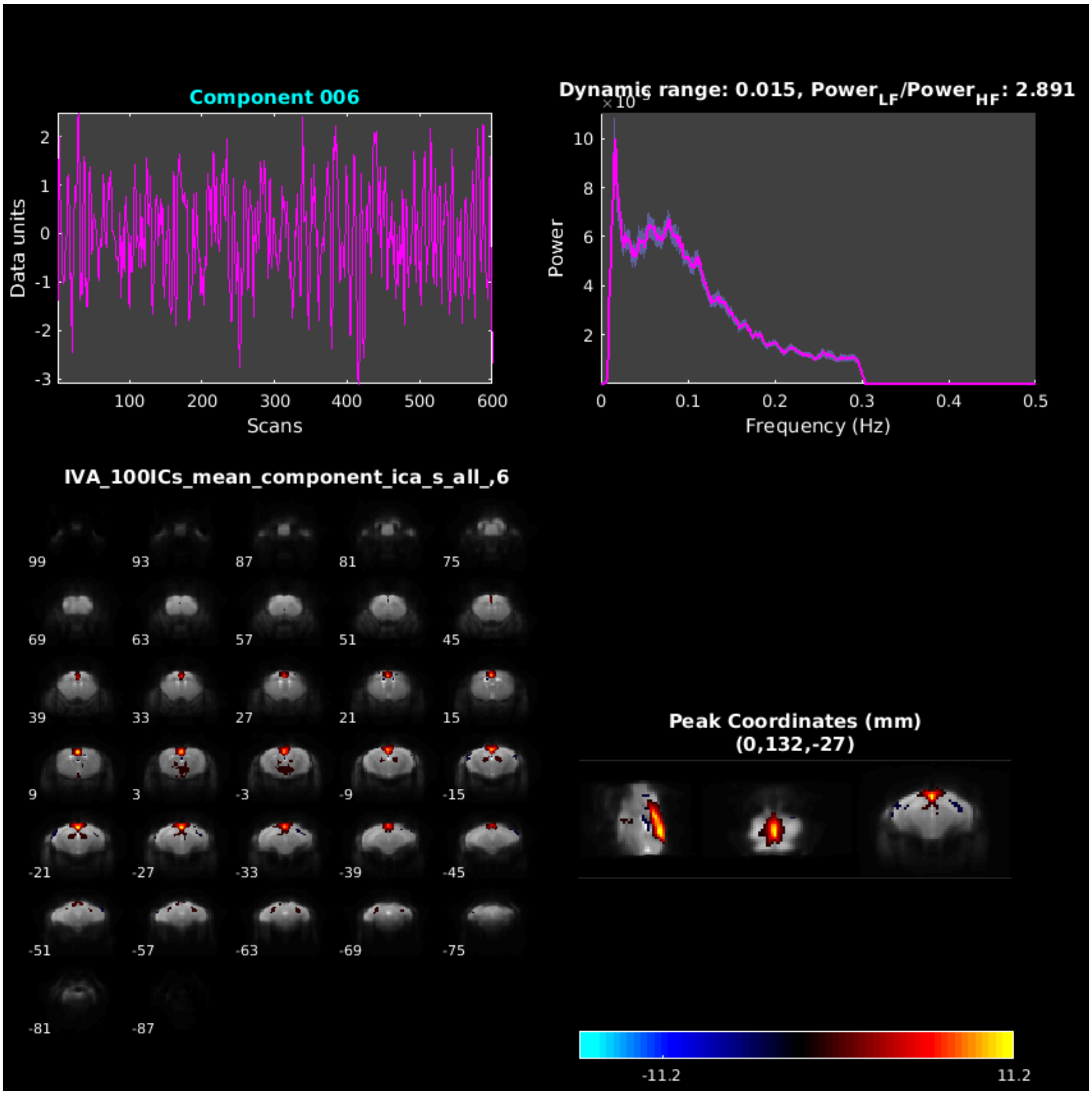

Retrosplenial area

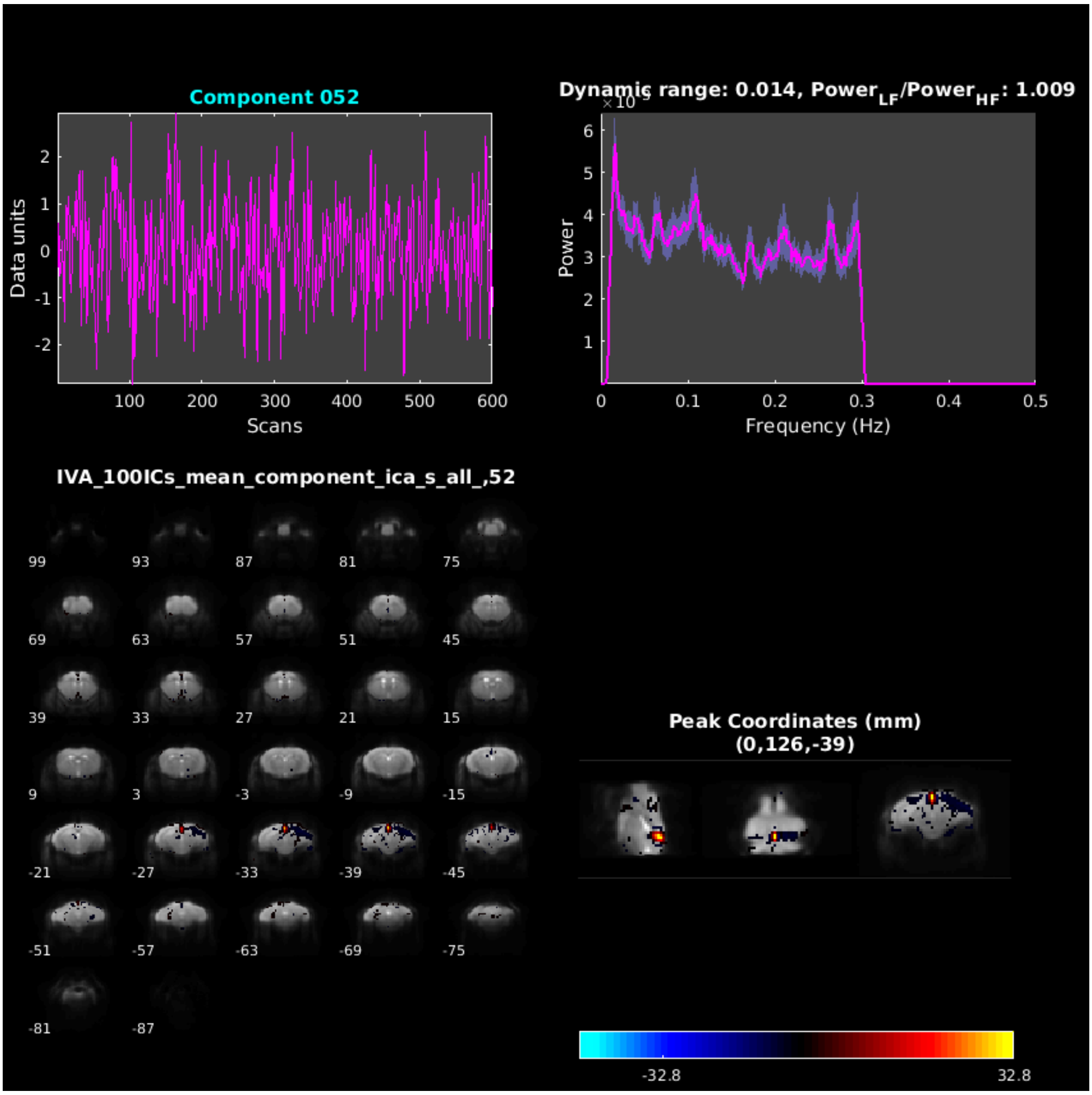

Anterior Cingulate Area

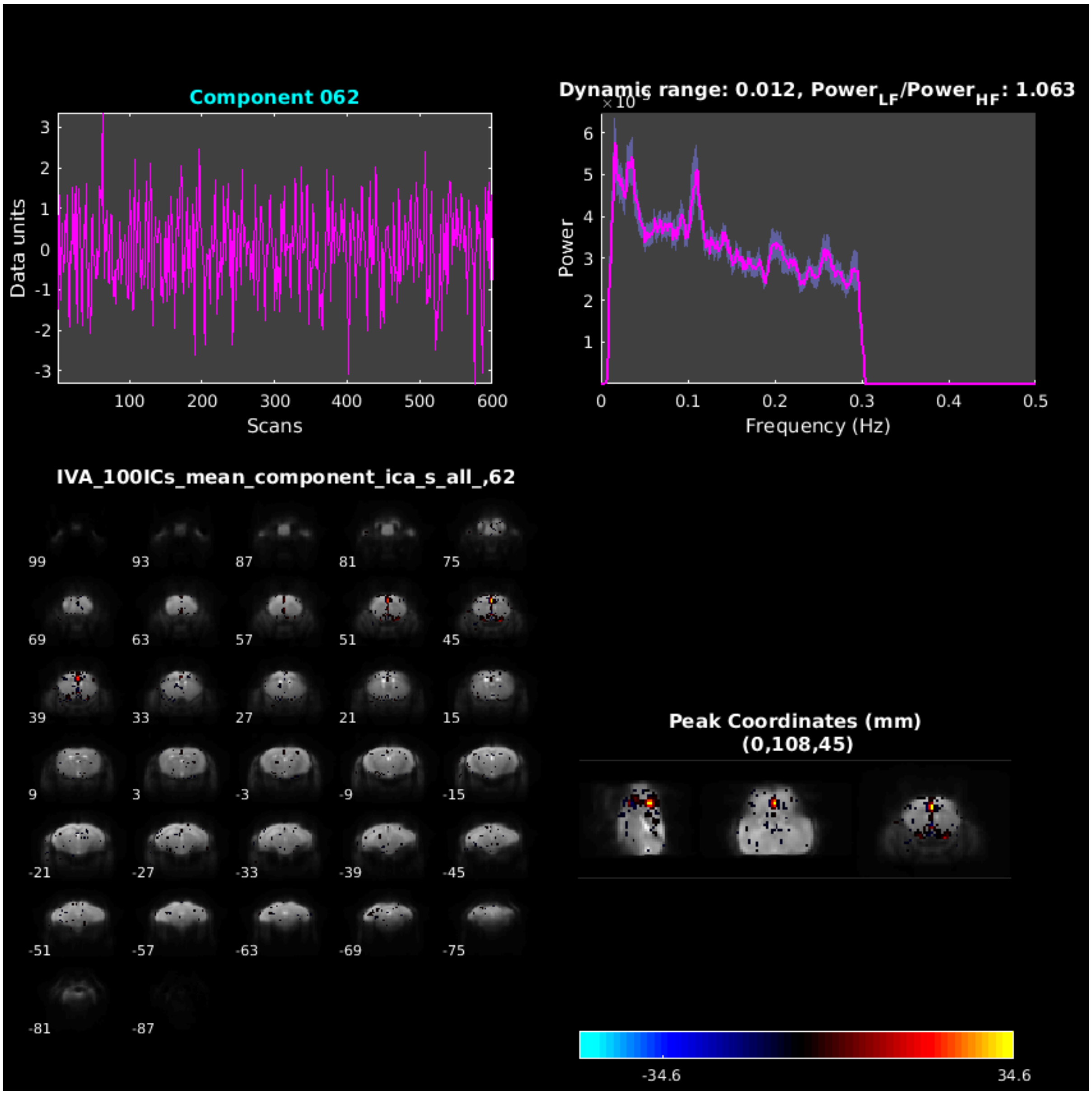

Retrosplenial area

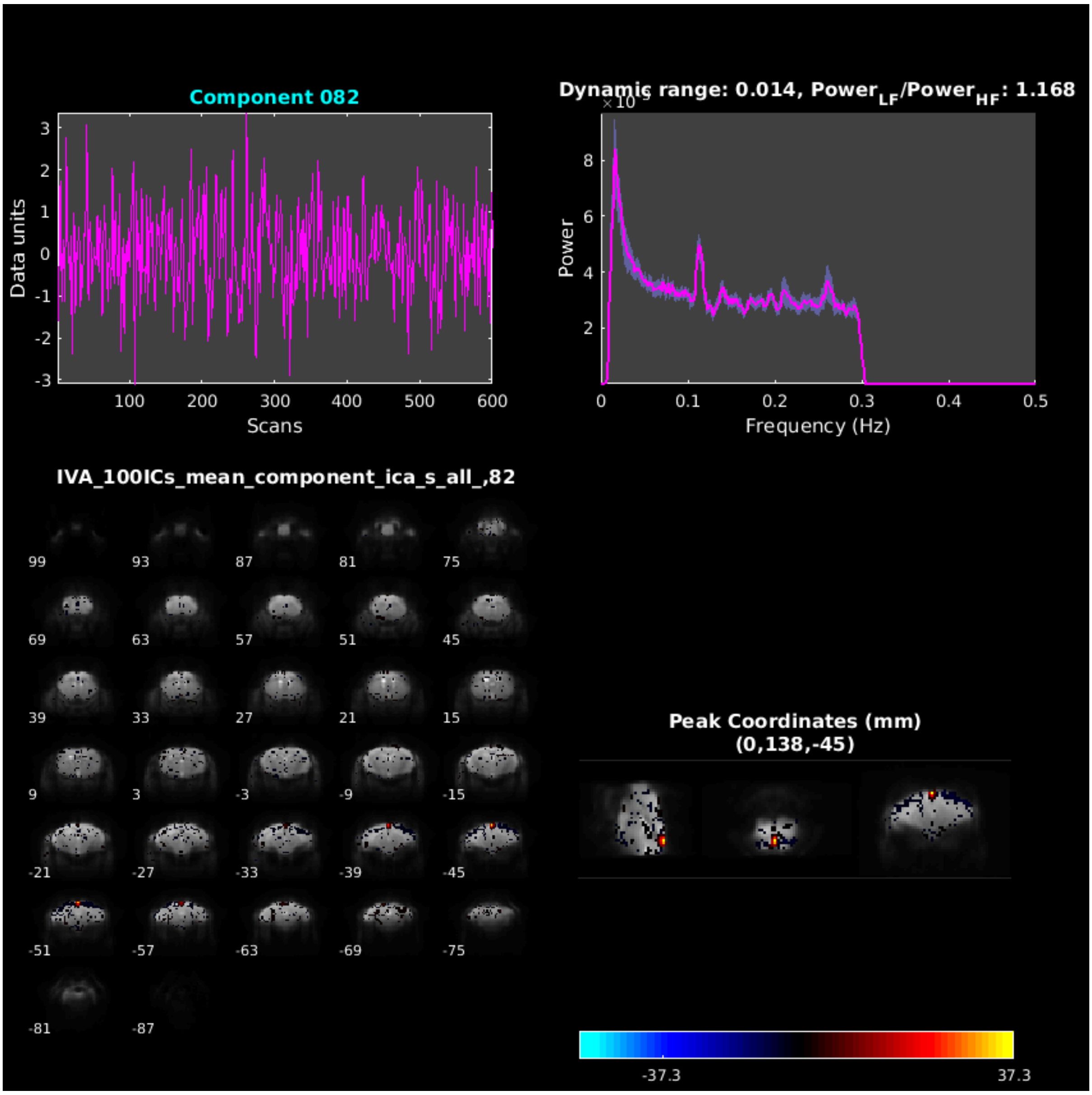

Infralimbic area

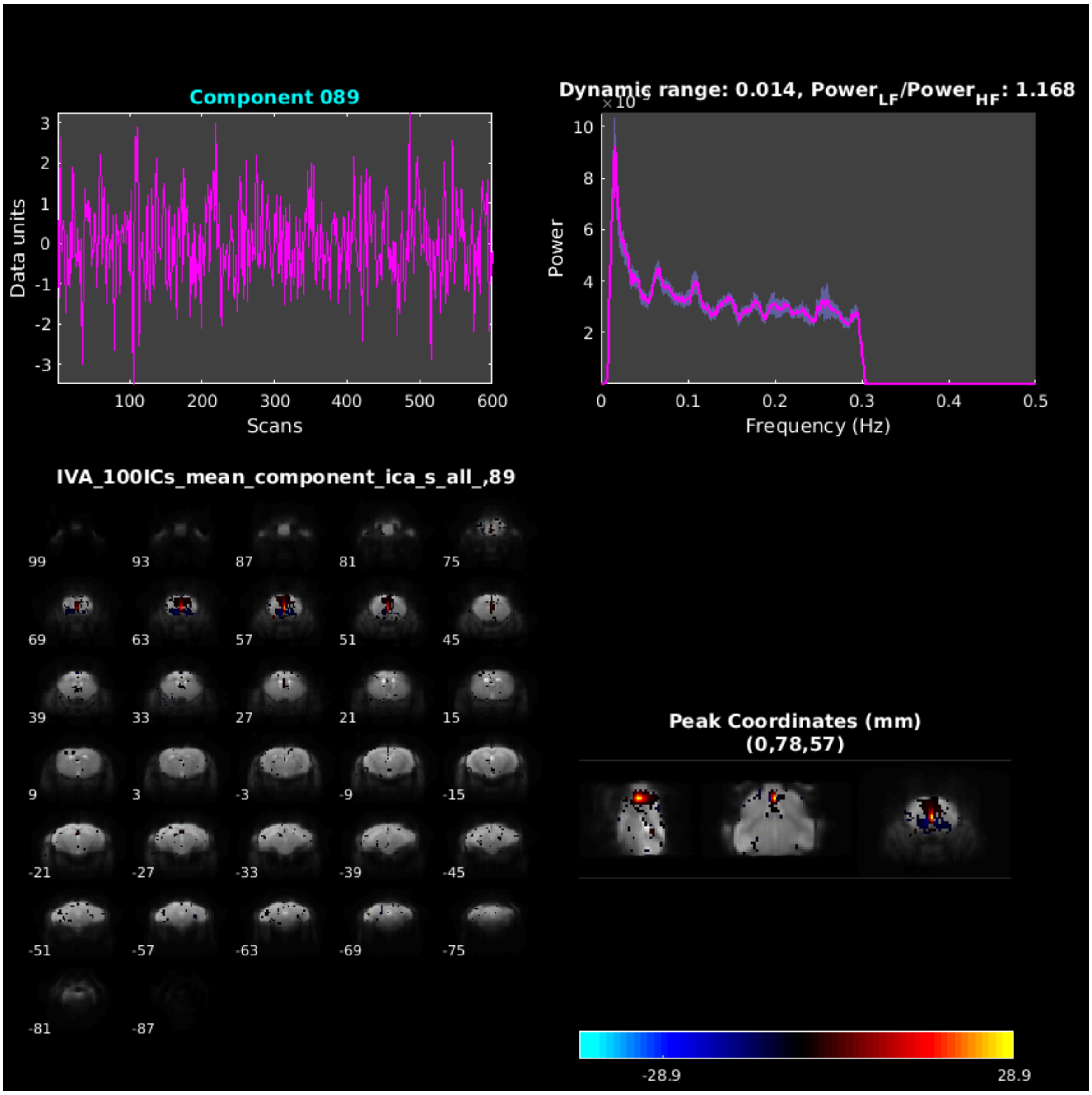

Subcortical spatial memory pathway (Retrohippocampal Area - Retrosplenial Area - Anterior Thalamic Nuclei - Anterior Cingulate Area)

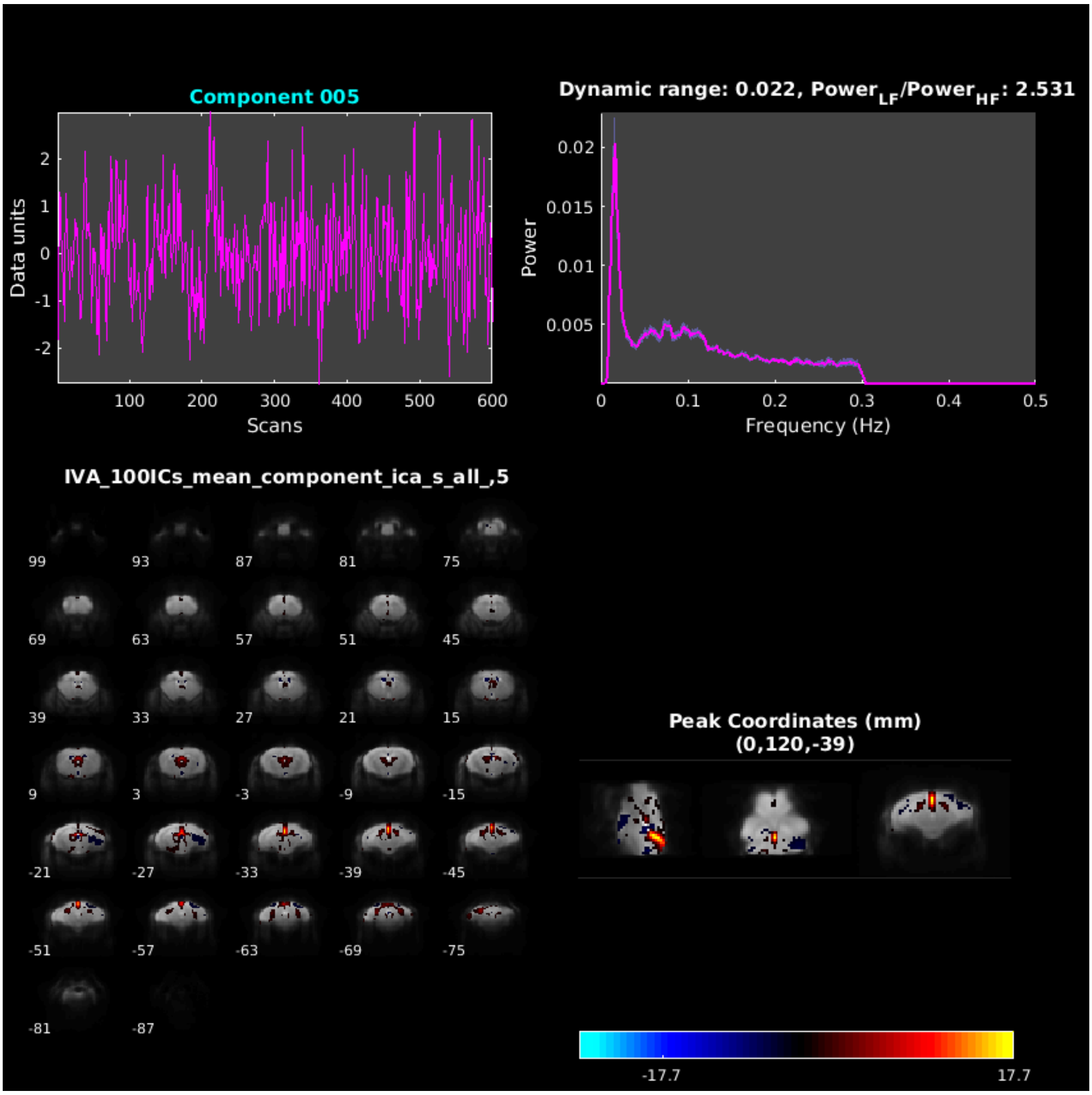

Dentate Gyrus

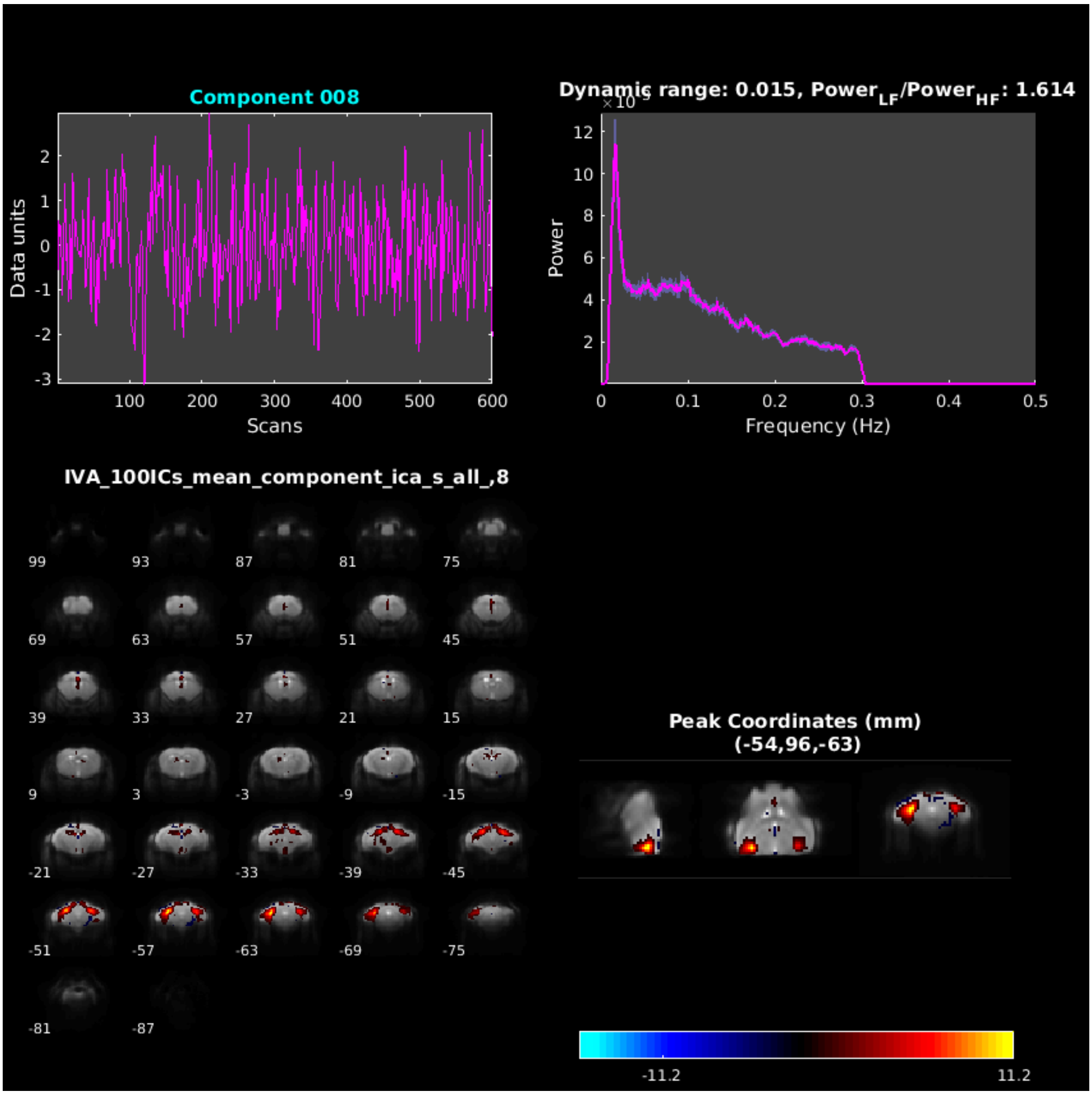

Anterior Thalamic Nucleus

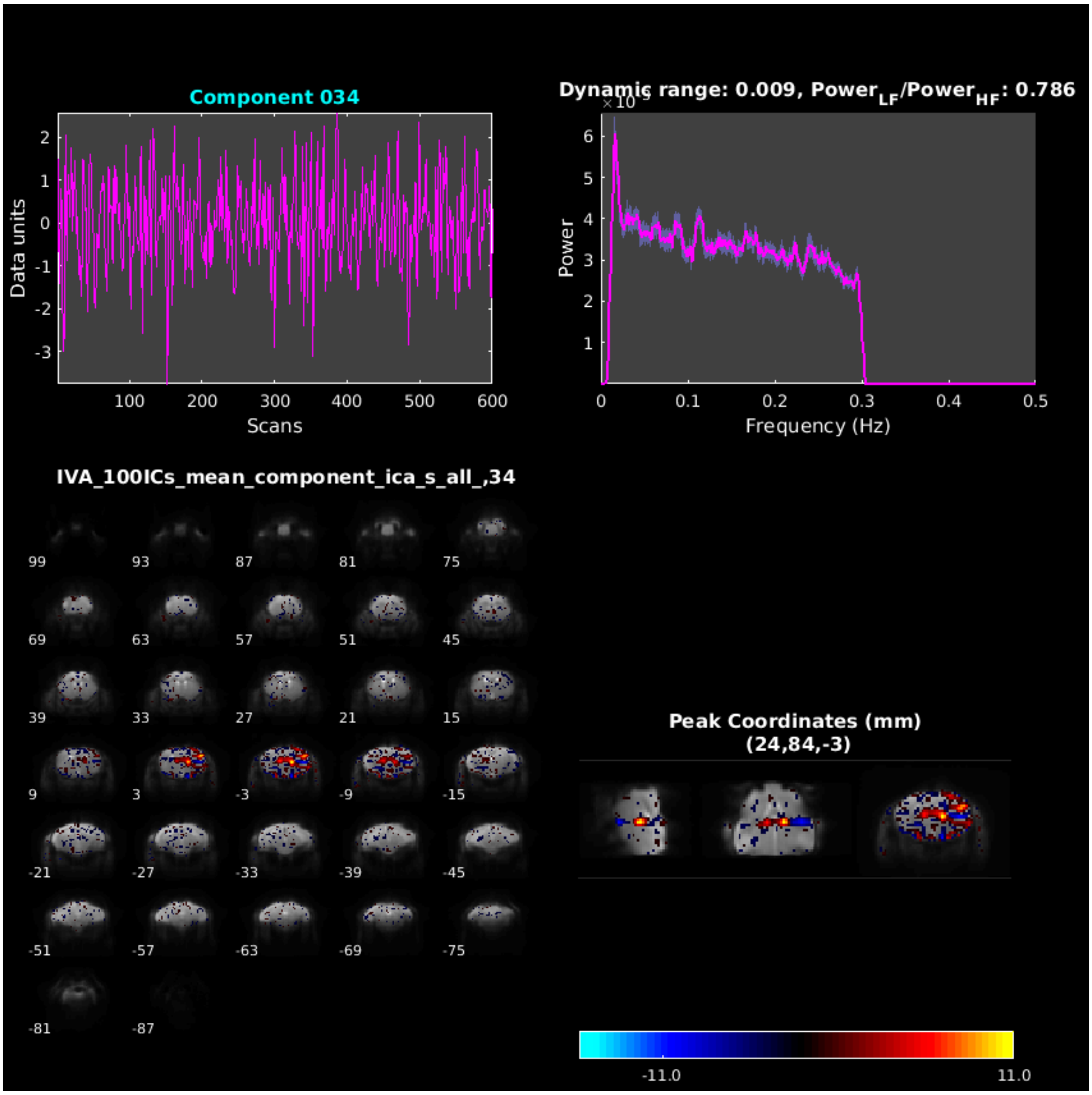

Auditory Area

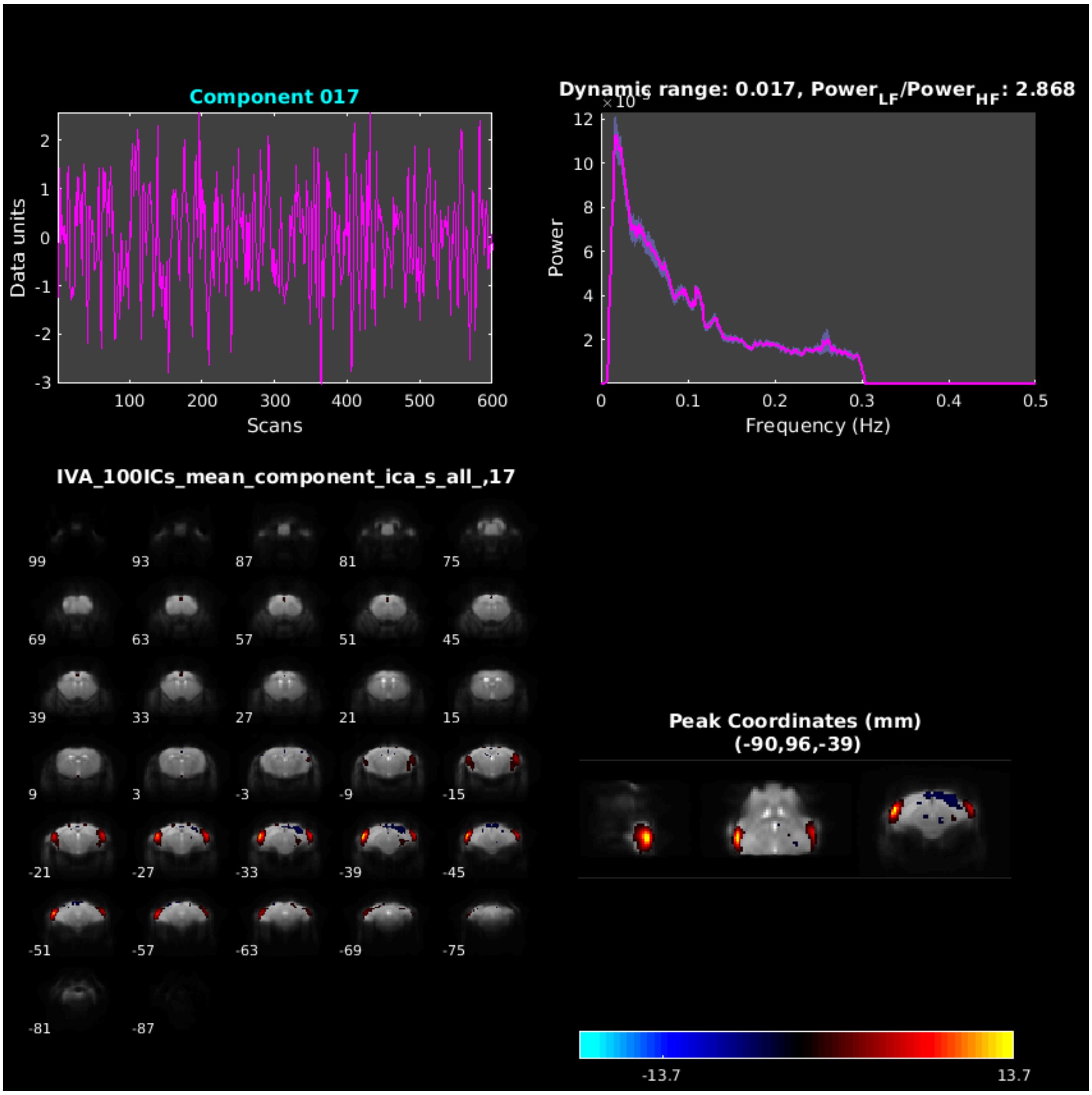

Visual Area

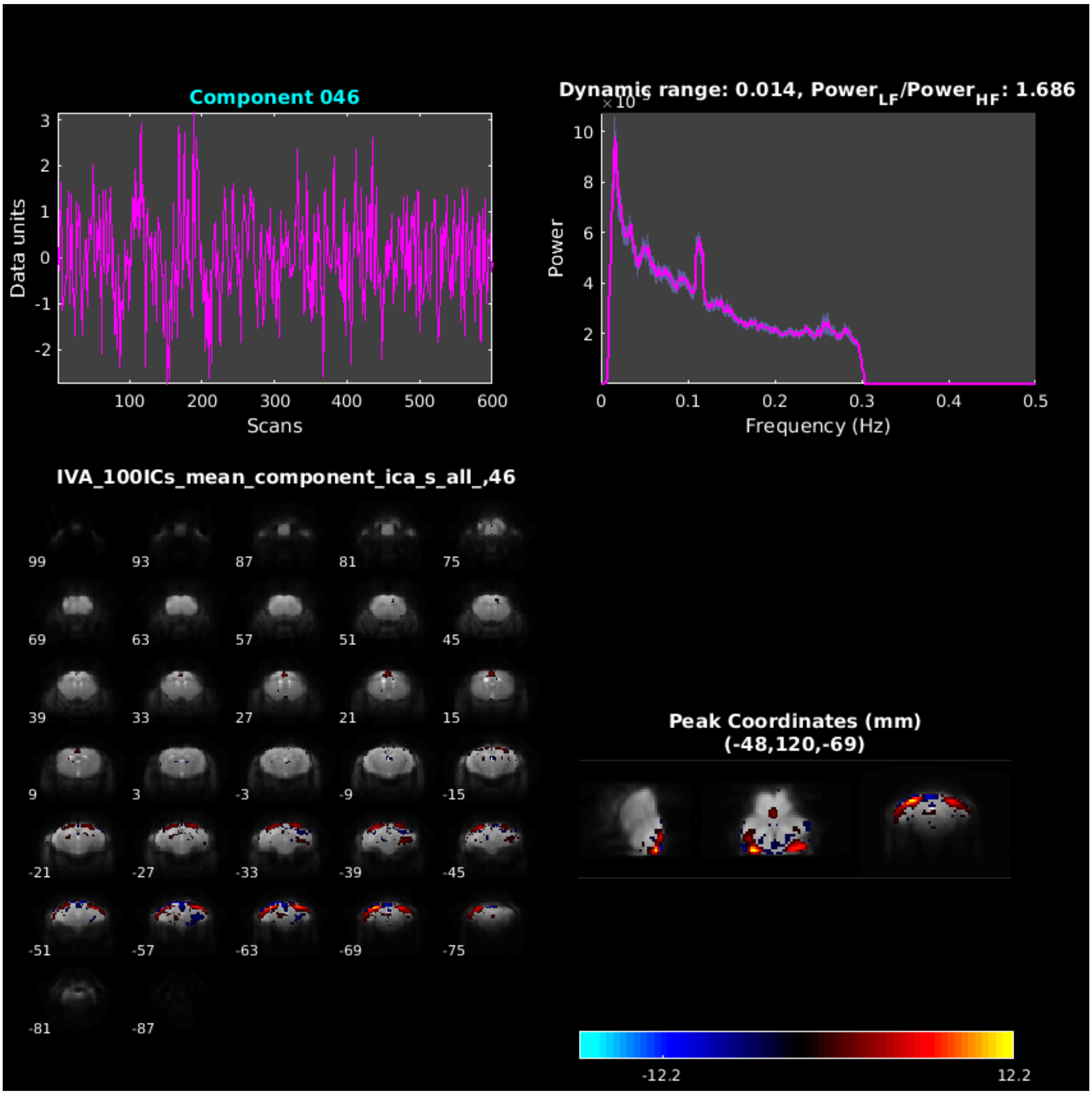

Component 046
Data units
Scans
Dynamic range: 0.014, Power$_{LF}$/Power$_{HF}$: 1.686
Power
Frequency (Hz)
IVA_100ICs_mean_component_ica_s_all_,46
Peak Coordinates (mm)
(-48,120,-69)
-12.2
12.2

Primary Somatosensory Cortex (S1) (Lower Limbs, Trunks) + Posterior Parietal Association Area + Temporal Association Area

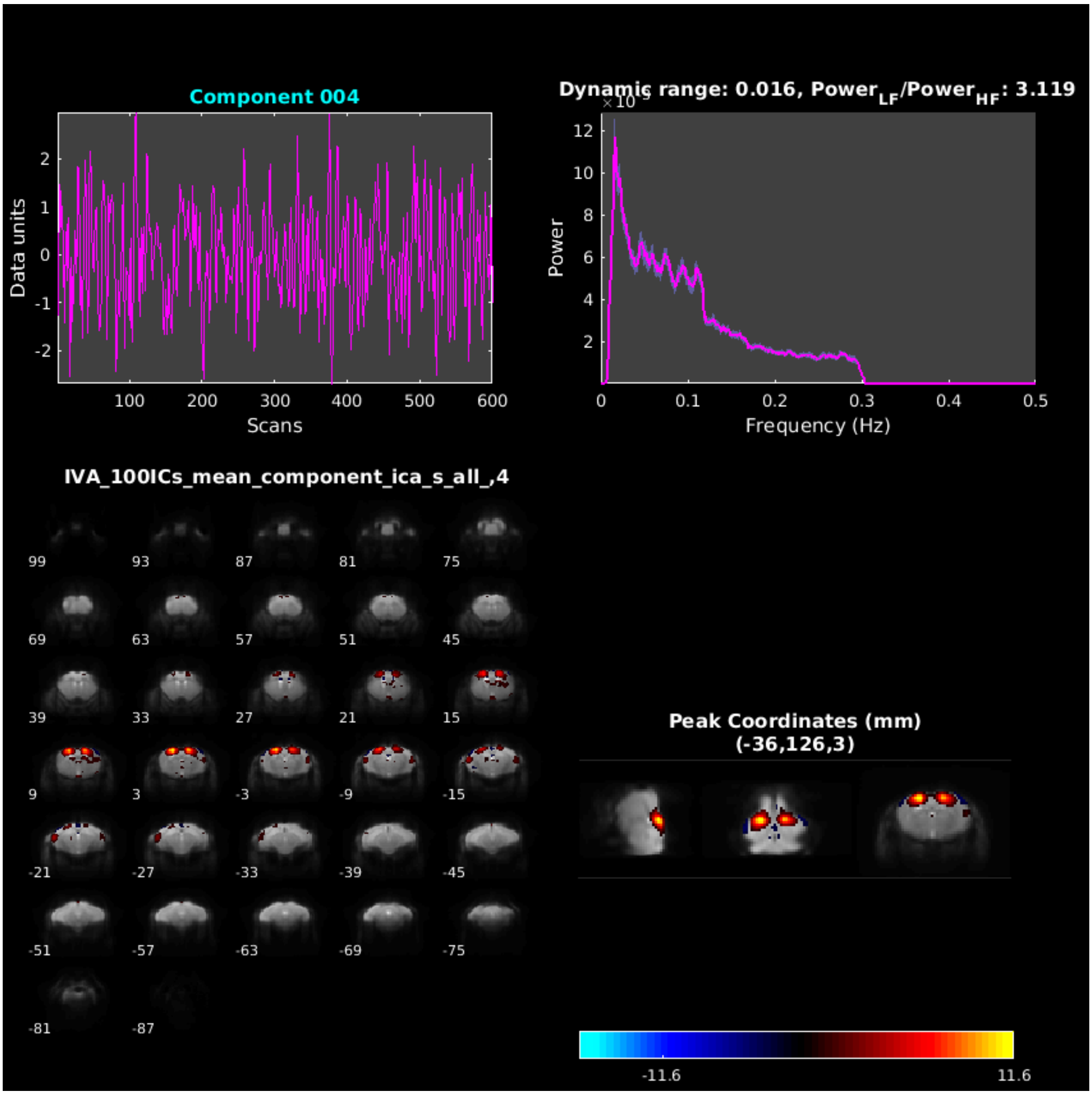

Primary Somatosensory Cortex (Upper Limbs)

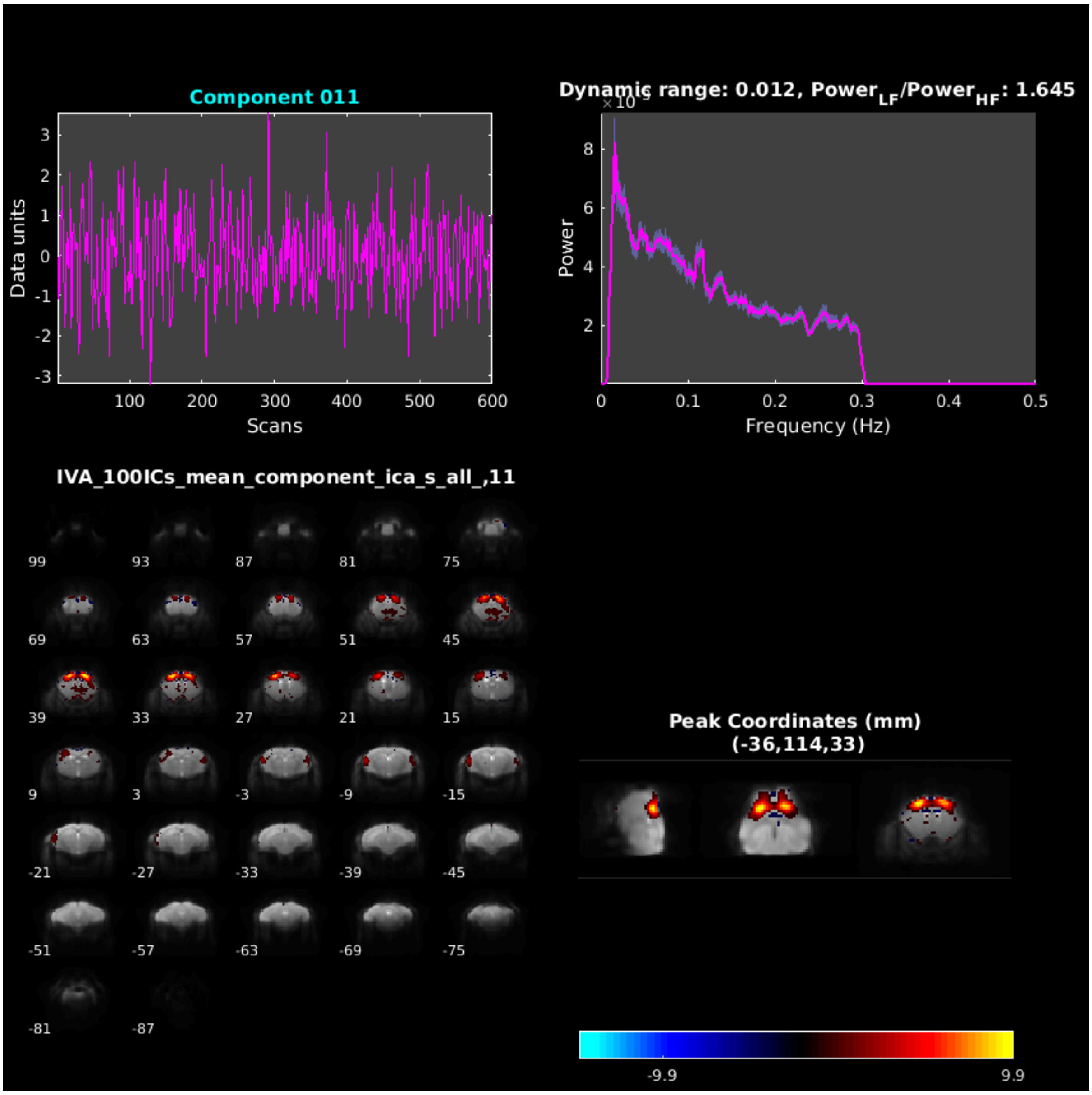

Primary Somatosensory Cortex (unassigned) + Visual Area

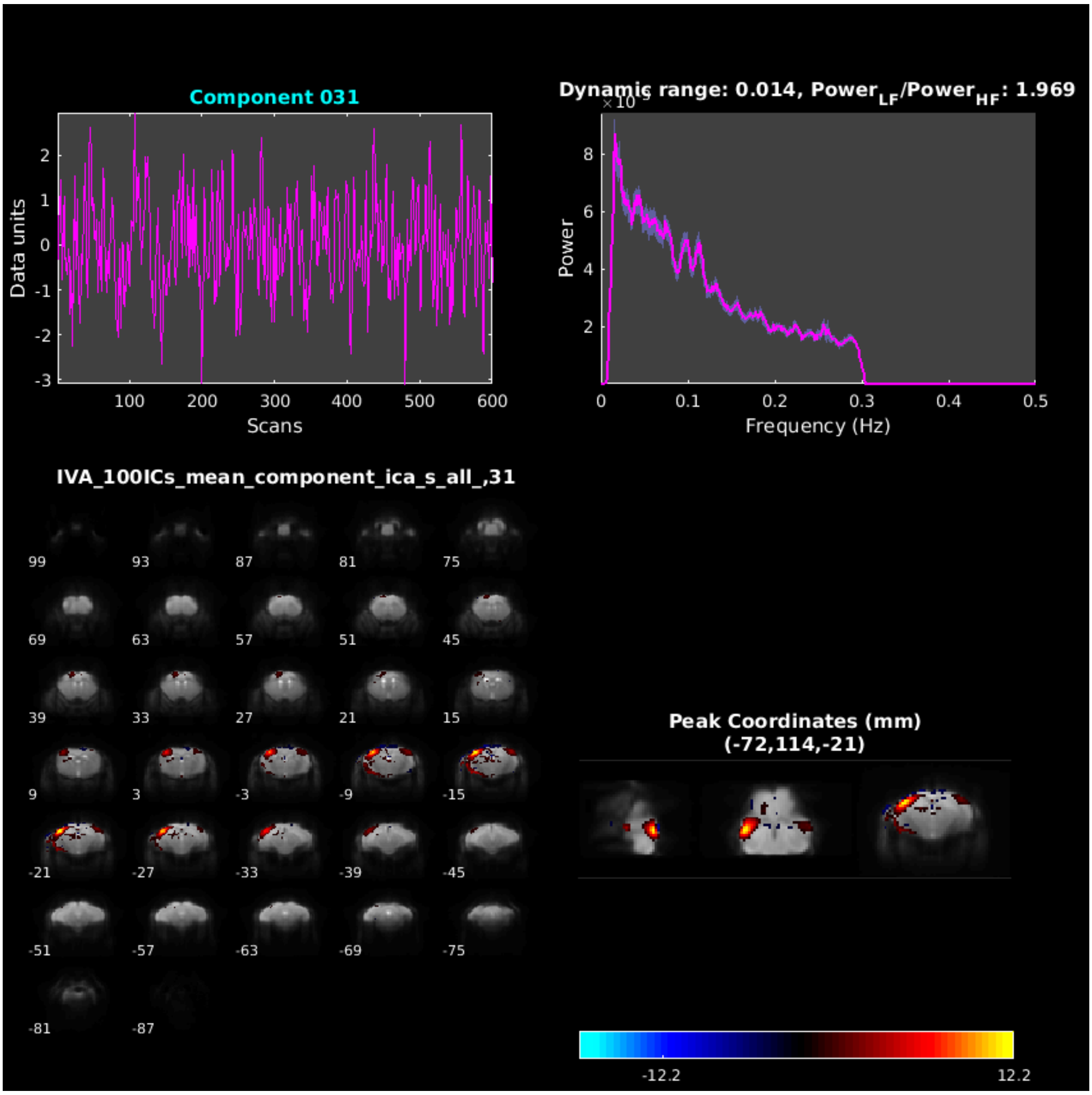

Thalamic nucleus (polymodal association cortex) + Primary Somatosensory Cortex

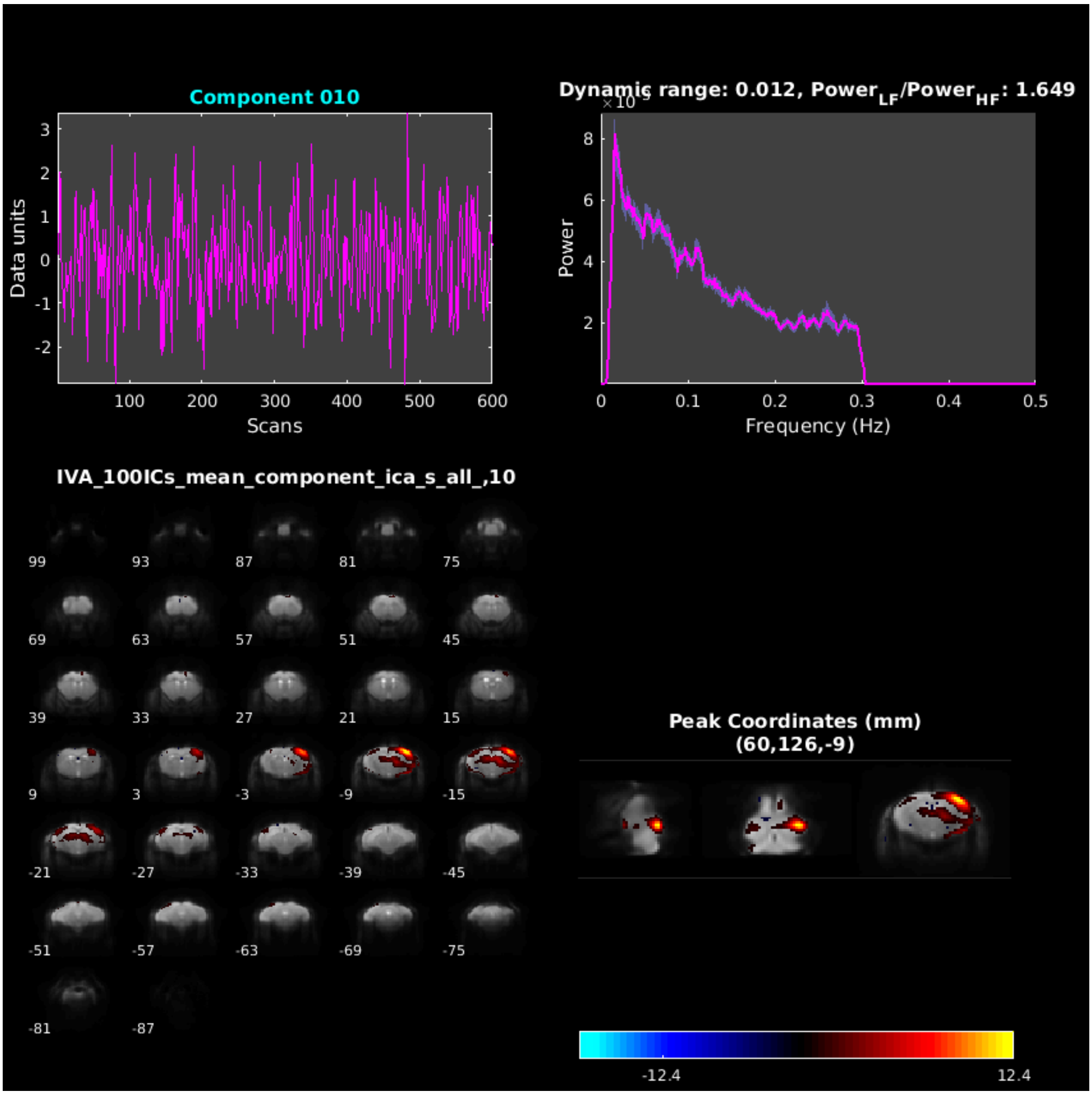

Thalamic nucleus (polymodal association cortex) + Pretectal Region + Visual-Auditory Area

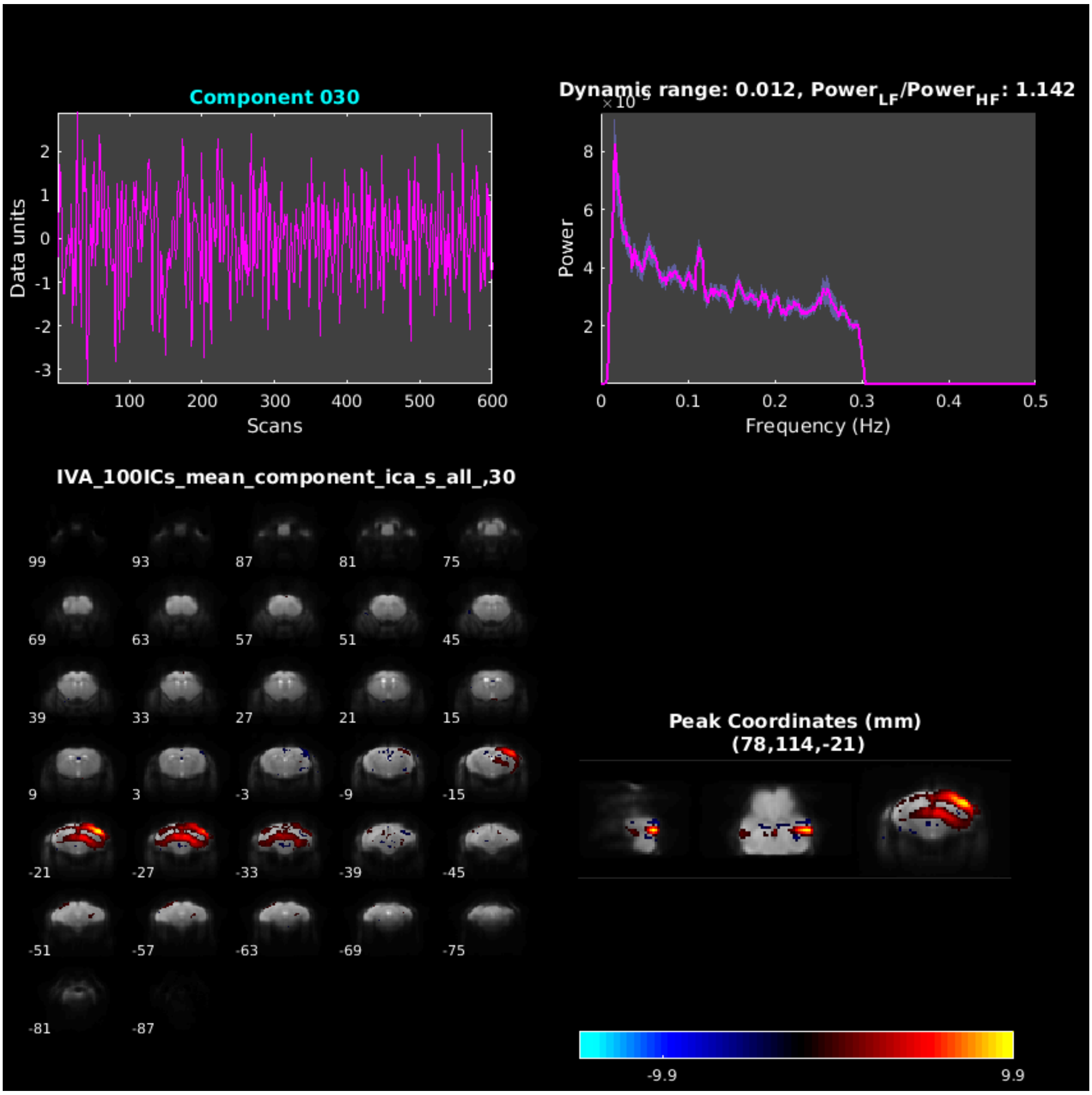

Caudate Putamen

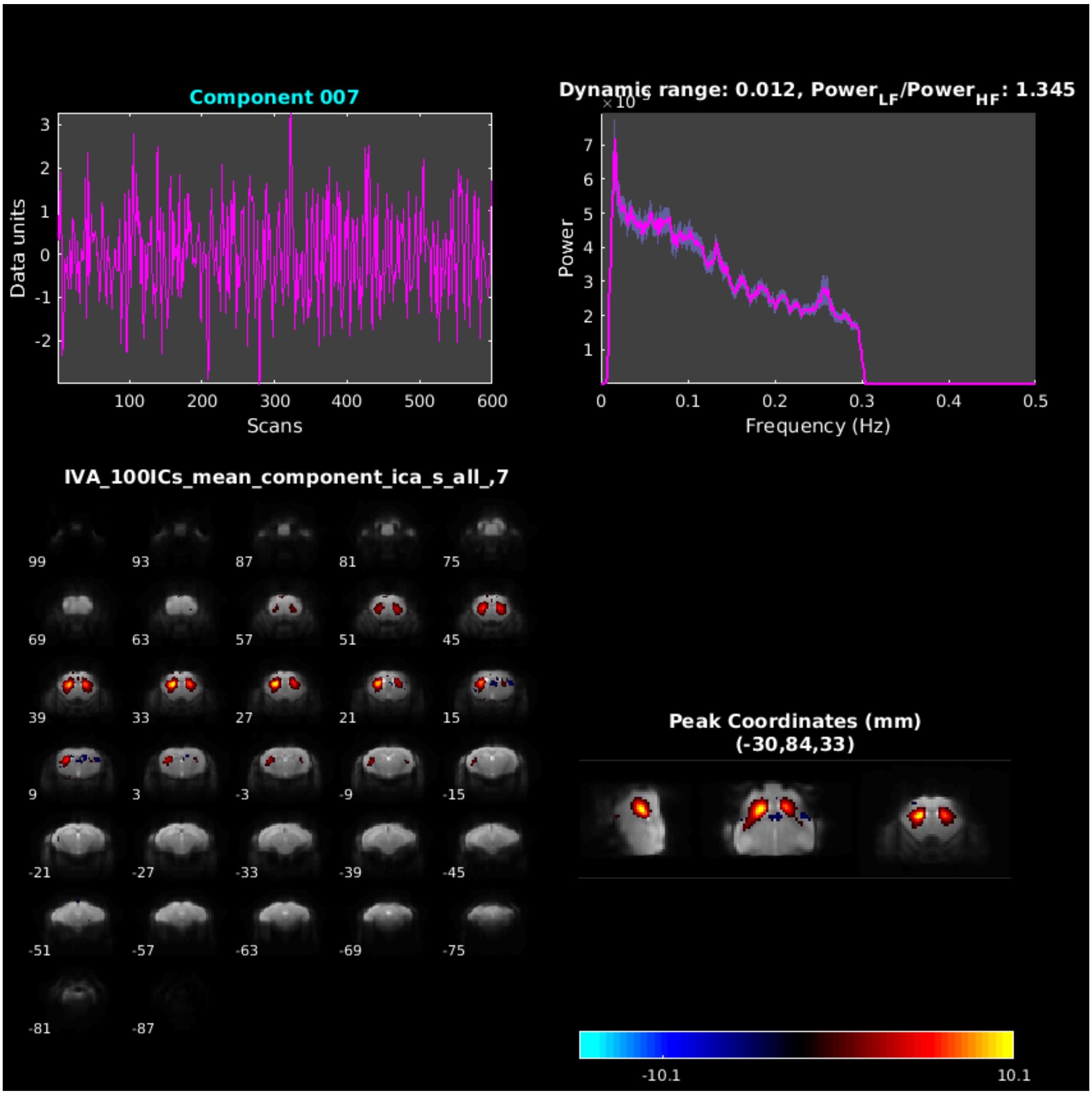

Primary Motor Cortex

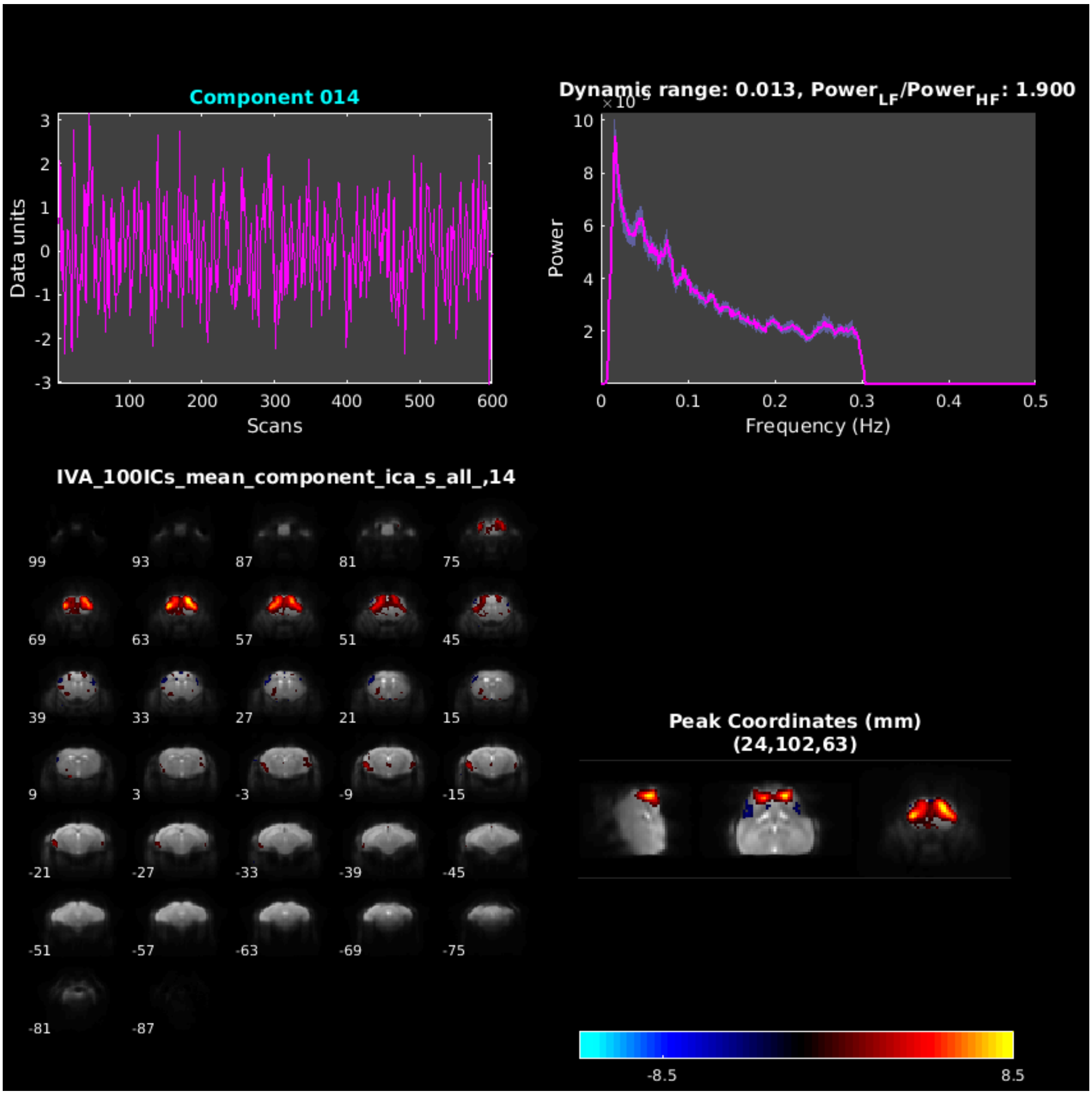

Primary Somatosensory Cortex (Barrel Field, Lower Limbs) + Primary Motor Area

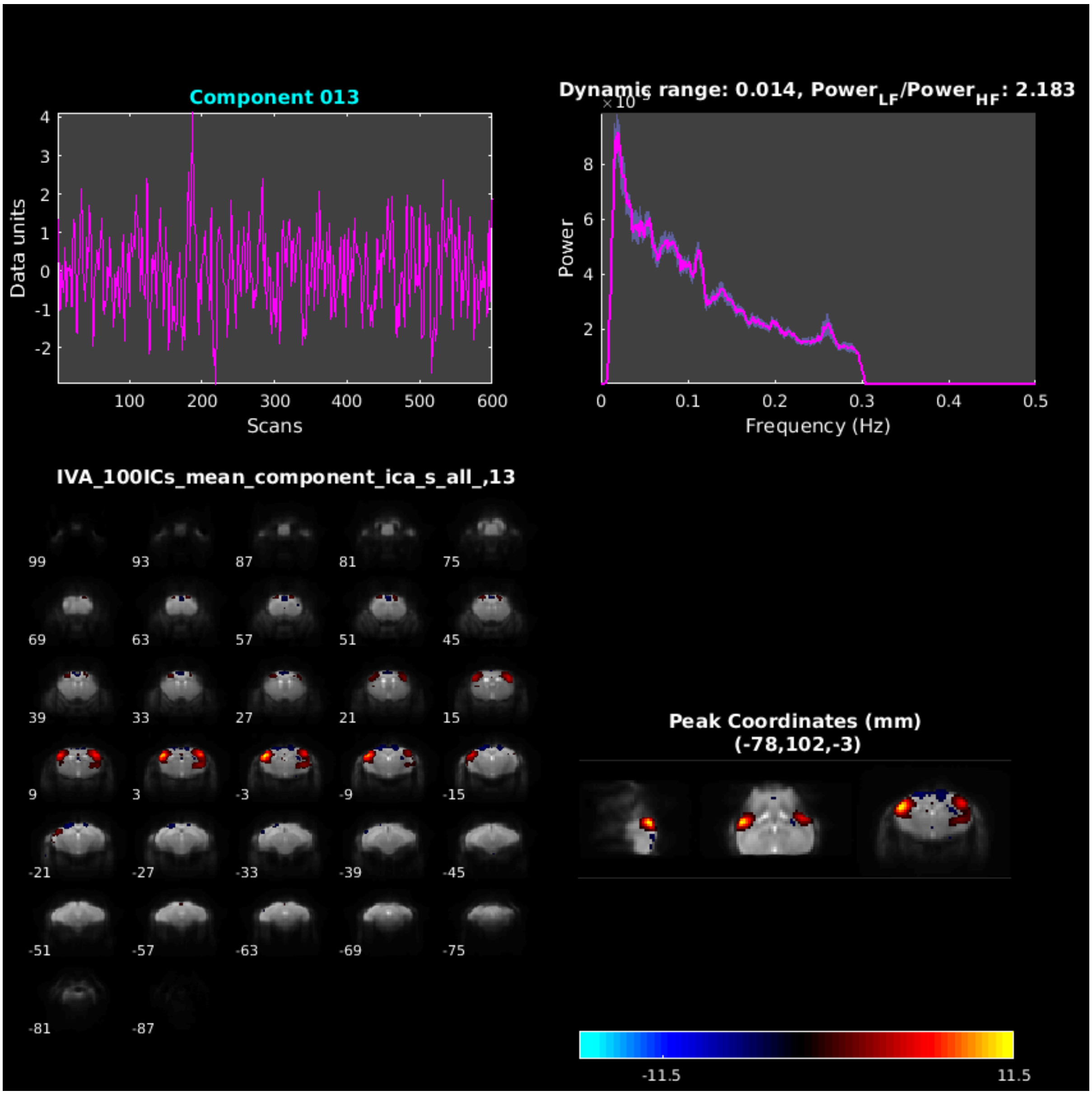

Insula + Endopiriform Nucleus

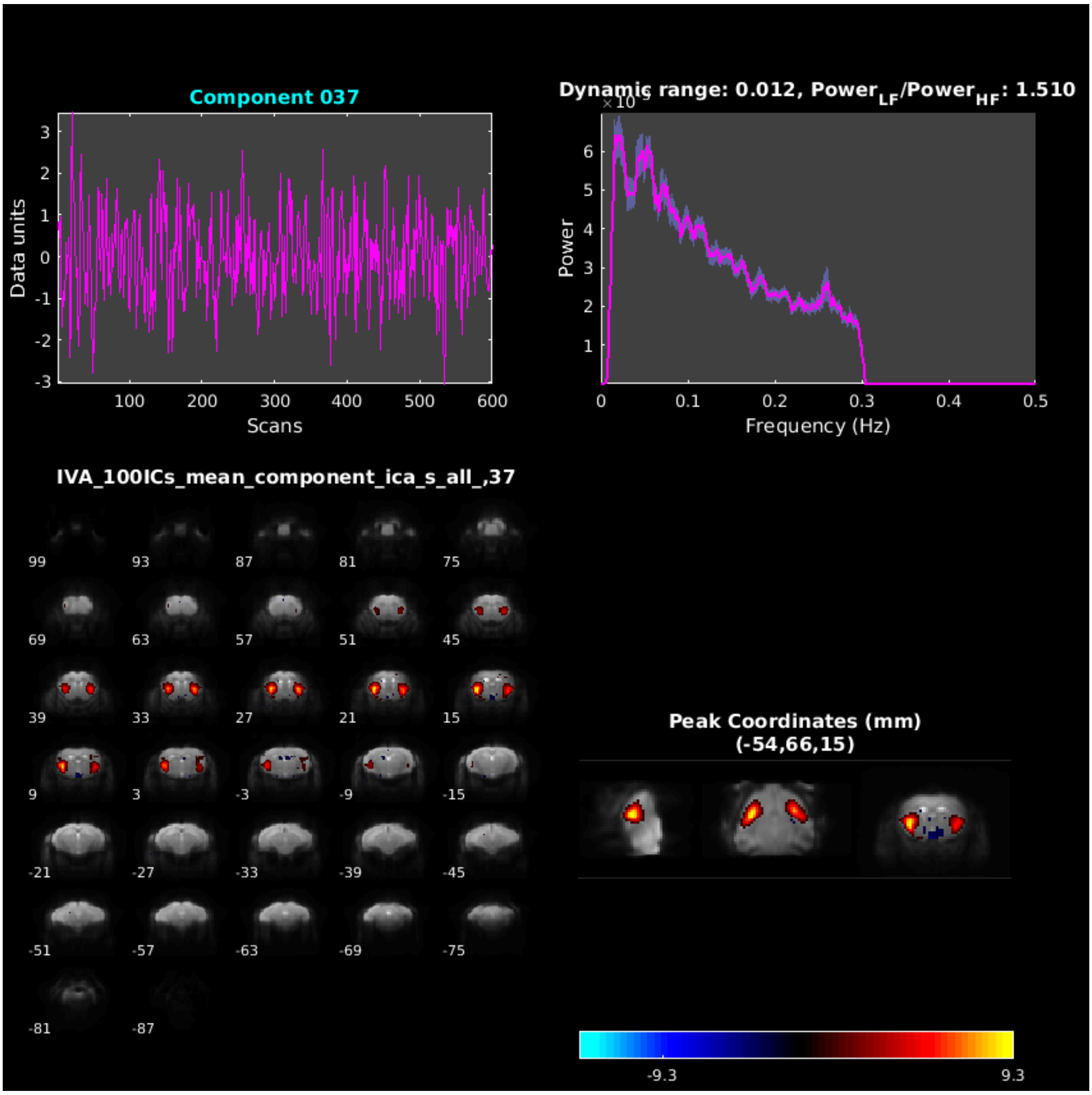

Amygdala

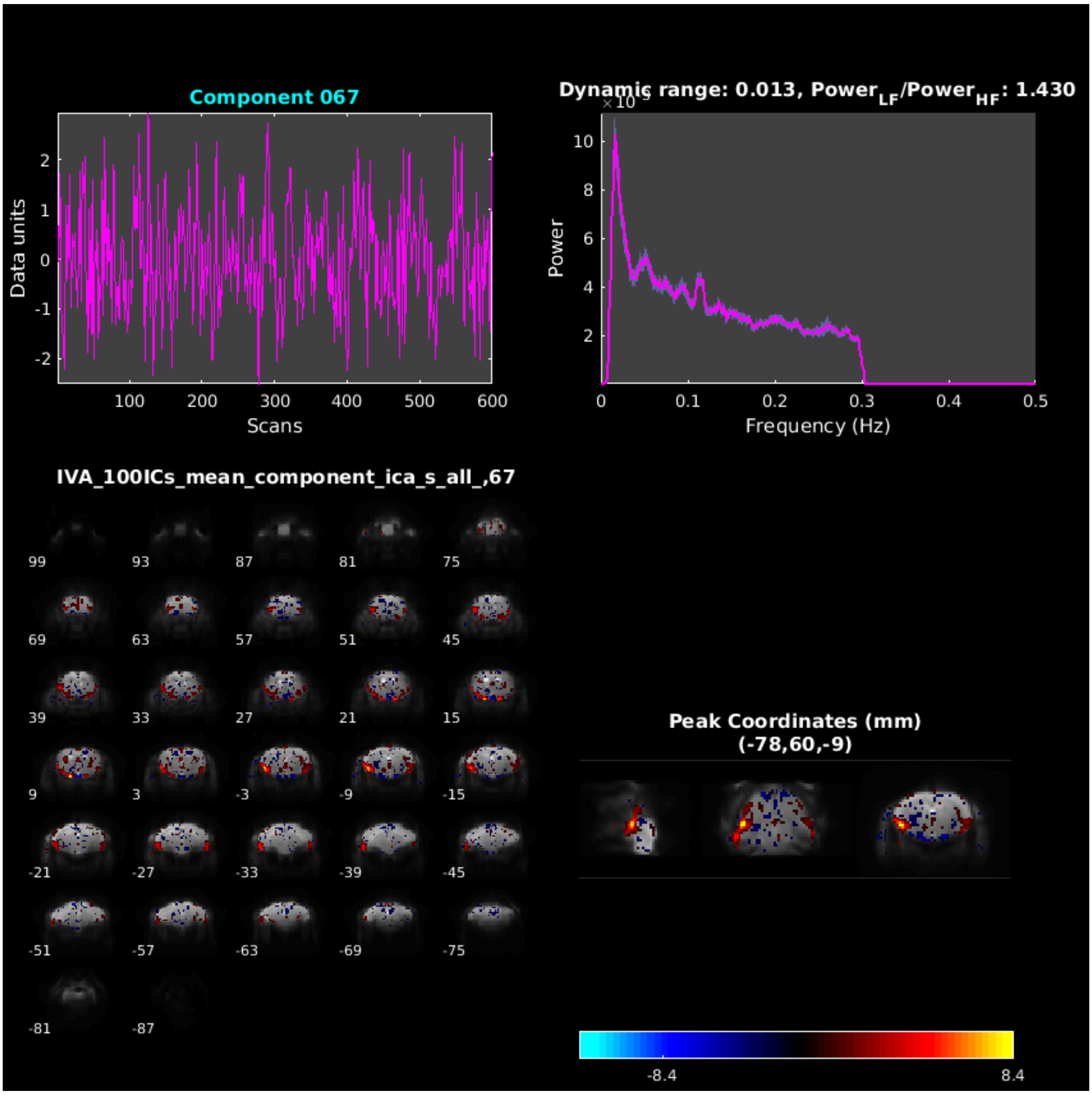

Component 067
Data units
Scans
Dynamic range: 0.013, Power_LF/Power_HF: 1.430
Power
Frequency (Hz)
IVA_100ICs_mean_component_ica_s_all_,67
Peak Coordinates (mm)
(-78,60,-9)
-8.4
8.4

Supplemental Somatosensory cortex (S2) - anterior (bi lateral), Primary Somatosensory Cortex (Nose, Mouth)

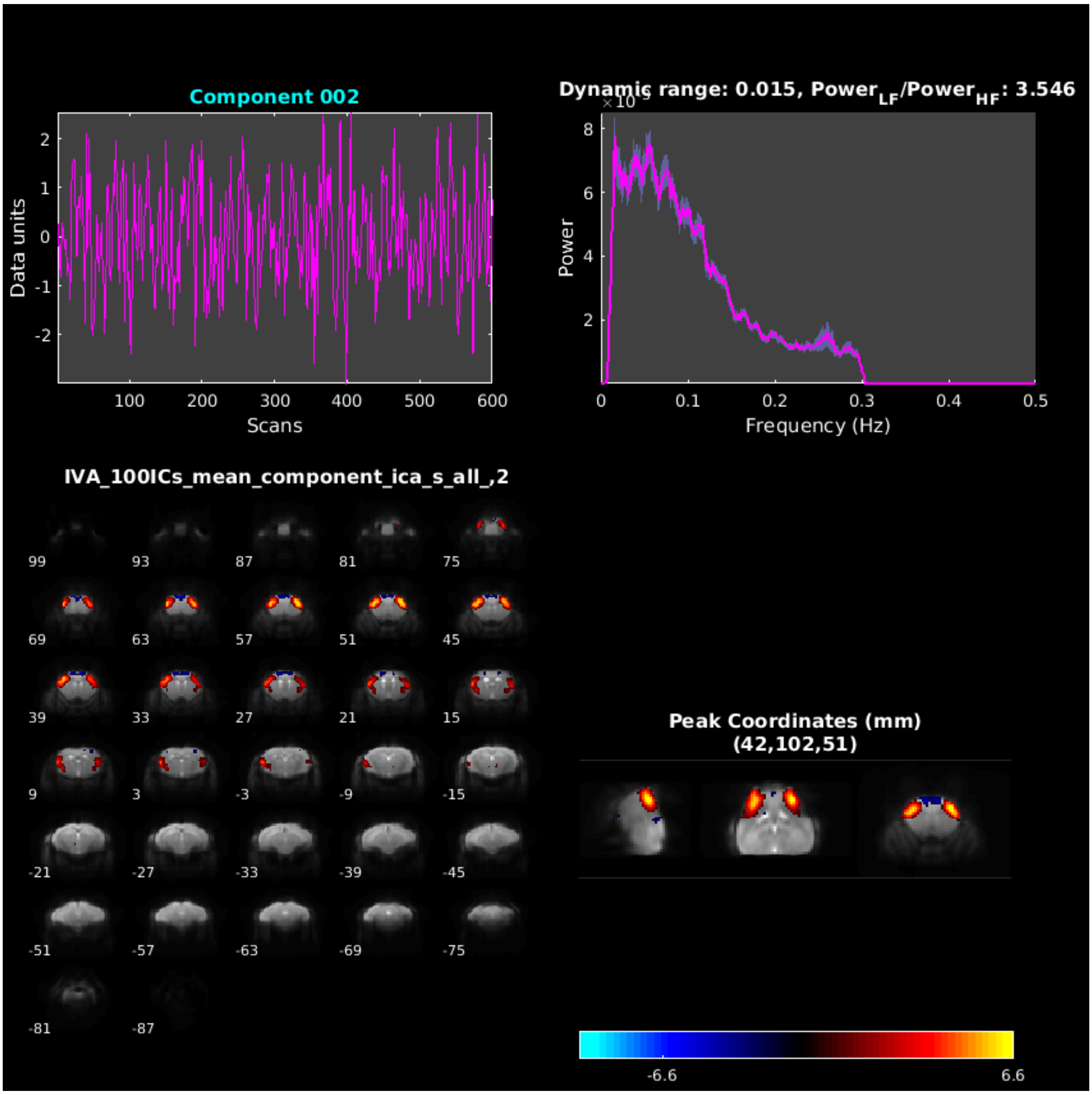

Supplemental somatosensory cortex – Right

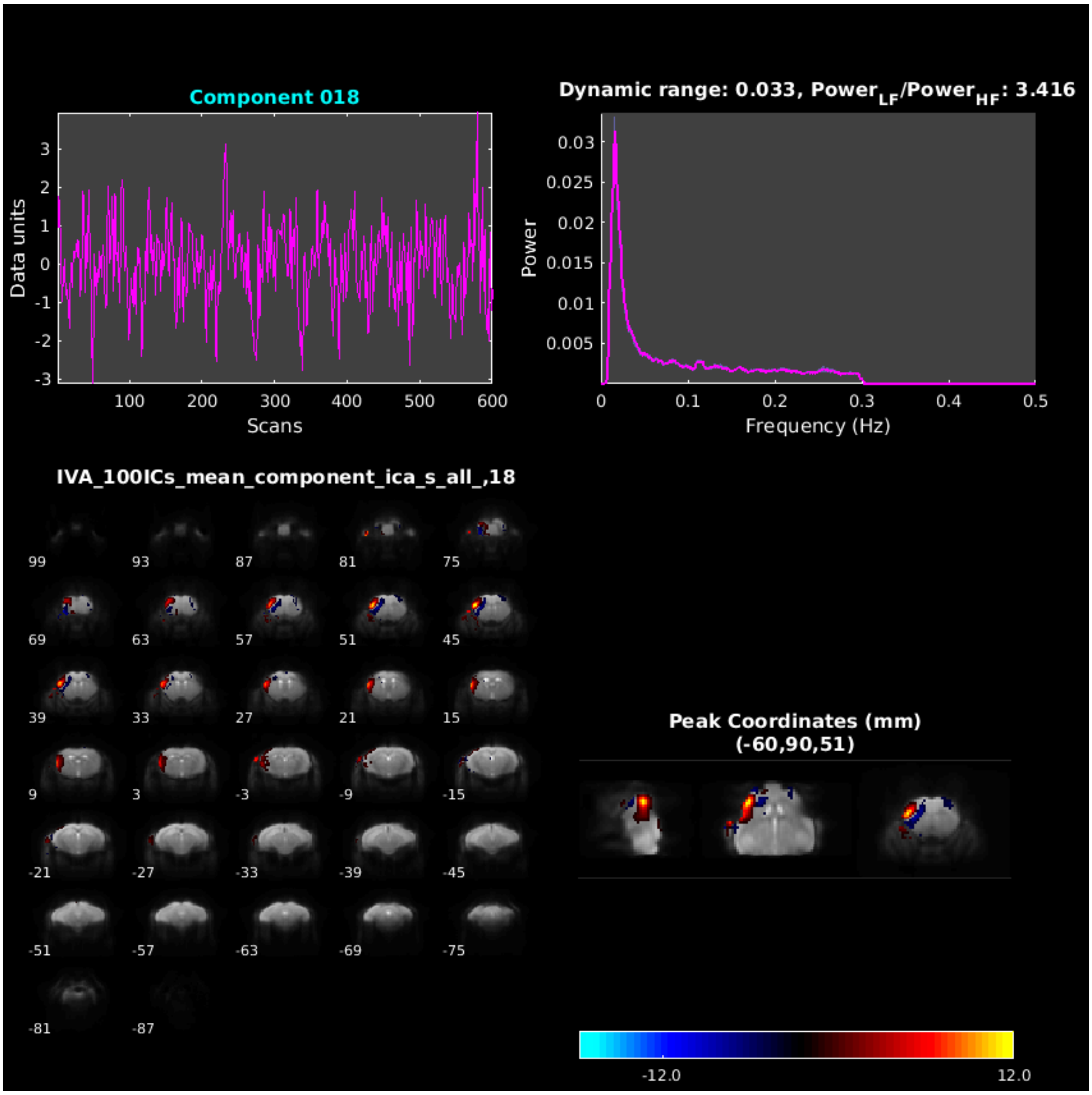

Supplemental somatosensory cortex – Left

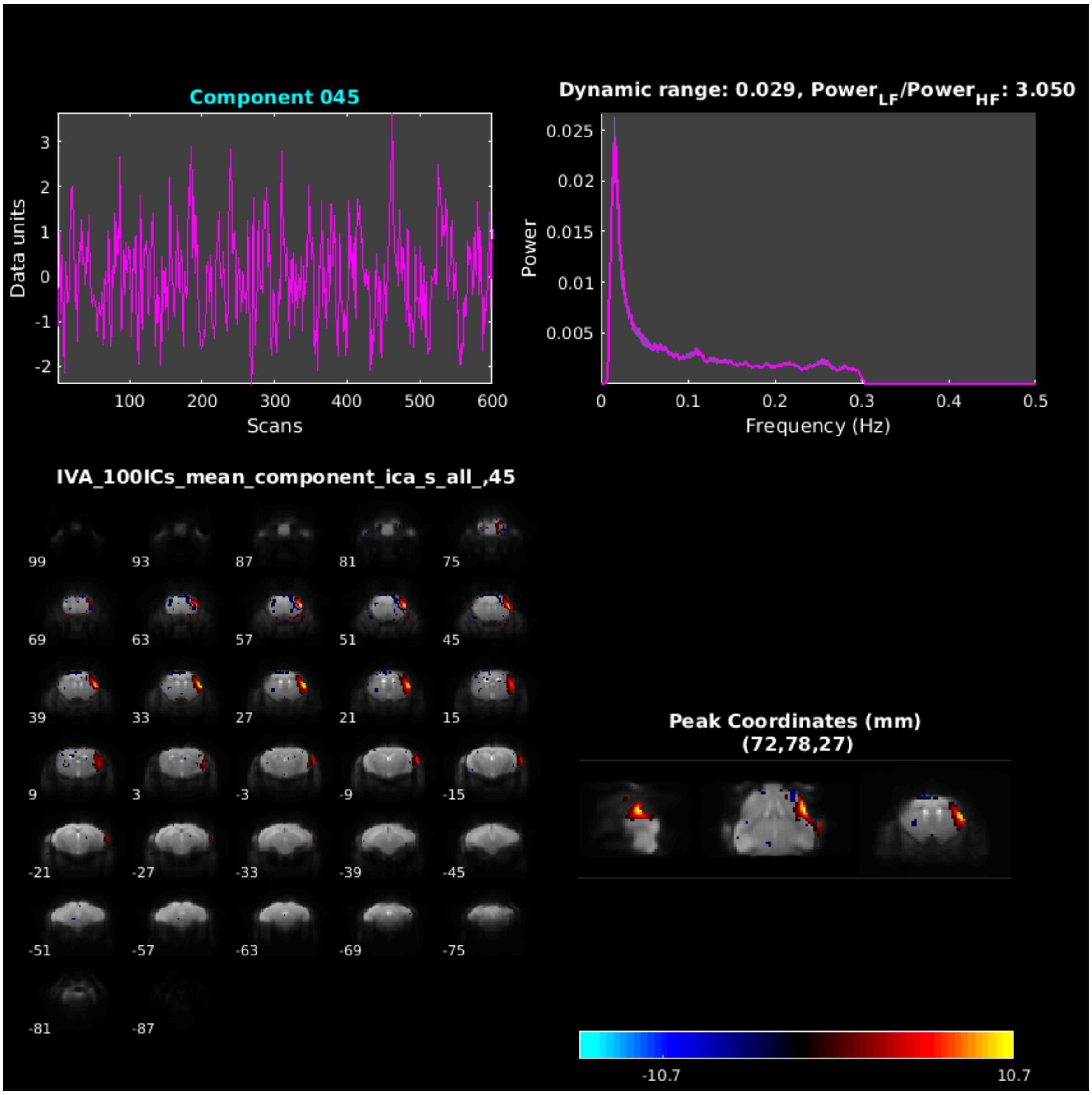

Supplement: Supplementary file 2 [file 41597_2021_985_MOESM2_ESM.pdf]
